# Materials to the revision of the genus *Cranichis* (Orchidaceae) in Bolivia

**DOI:** 10.3897/phytokeys.186.71499

**Published:** 2021-11-29

**Authors:** Marta Kolanowska, Przemysław Baranow, Sławomir Nowak, Alfredo Fuentes

**Affiliations:** 1 University of Lodz, Faculty of Biology and Environmental Protection, Department of Geobotany and Plant Ecology, Lodz, Poland University of Lodz Lodz Poland; 2 Department of Biodiversity Research, Global Change Research Institute AS CR, Brno, Czech Republic Department of Biodiversity Research, Global Change Research Institute AS CR Brno Czech Republic; 3 Department of Plant Taxonomy and Nature Conservation, University of Gdańsk, Gdańsk, Poland University of Gdańsk Gdańsk Poland; 4 Herbario Nacional de Bolivia, Instituto de Ecología, Universidad Mayor de San Andrés, La Paz, Bolivia Universidad Mayor de San Andrés La Paz Bolivia

**Keywords:** Cranichidinae, diversity, new species, taxonomy

## Abstract

The diversity of *Cranichis* in Bolivia is evaluated. An updated key for identifying species is provided. Morphological characteristics of 15 species of Bolivian *Cranichis* are presented together with illustrations of their floral segments. The occurrence of *C.diphylla*, *C.lehmannii*, and *C.muscosa* in this country was not confirmed. In our opinion the previously published Bolivian record for *C.polyantha* is doubtful. For the first time, *C.badia* and *C.longipetiolata* are reported in this country. Two new species of *Cranichis* are described.

## ﻿Introduction

Bolivian Orchidaceae are the least studied in terms of biodiversity (Vásquez et al. 2003), however, novelties are being reported (e.g. Dalström 2006; Pupulin and Moreno 2018; Kolanowska et al., 2019; Pace 2020). A preliminary revision of Bolivian material has revealed some interesting discoveries within the Cranichidinae (Kolanowska et al. 2020), which are a significant element of the terrestrial neotropical flora (Salazar et al. 2009).

According to Vásquez et al. (2014) there are six genera of Cranichidinae*sensu*Dressler (1993; *Baskervilla* Lindl., *Cranichis* Sw., *Ponthieva* R.Br., *Pseudocentrum* Lindl., *Pterichis* Lindl., and *Solenocentrum* Schltr.) and six of Prescottiinae (Dressler 1990; *Aa* Rchb.f., *Altensteinia* Kunth, *Gomphichis* Lindl., *Myrosmodes* Rchb.f., *Prescottia* Lindl., and *Stenoptera* C.Presl.) present in Bolivia.

*Cranichis* was described by Swartz in 1788 and typified with *C.muscosa* Sw. over 150 years later by Acuña (1939). Species of *Cranichis* are usually terrestrial or lithophilic plants characterized by petiolate leaves, non-resupinate ﬂowers, petals much narrower than the sepals, and cochleate lip that is often conspicuously veined or ornamented with nodules. The gynostemium of *Cranichis* is massive, often swollen at the apex with thick, massive, spacious clinandrium and elongated, digitate, thick hamulus (Szlachetko and Rutkowski 2000; Kolanowska and Szlachetko 2015). The species grow in various habitats at altitudes ranging from 350 to over 3000 m (Carnevali and Ramírez-Morillo 2003; Cribb 2003). The geographical range of *Cranichis* extends from USA (Florida) south to Bolivia and Argentina.

Vásquez et al. (2014) report nine species of *Cranichis* occurring in Bolivia and listed *C.castellanosii* L.O. Williams as unconfirmed taxon. However, *C.fertilis* (F. Lehm. & Kraenzl.) Schltr. catalogued by the authors, has been earlier included (as a synonym of *Ophrysparviflora* Presl) in the genus *Exalaria* Garay & G.A. Romero (Garay and Romero 1999). Moreover, Vásquez et al. (2014) accepted the broad concept of *C.ciliata* (Kunth) Kunth and *C.diphylla* Sw. In this recognition, the authors included several synonyms of the former species (e.g. *C.atrata* Schltr., *C.pleioneura* Schltr., *C.polyblephara* Schltr., *C.sororia* Schltr., and *C.mandonii* Schltr). *Cranichisnigrescens* Schltr., *C.ovatilabia* Schltr., and *C.stictophylla* Schltr. are accepted as synonyms of *C.diphylla.* In addition, three species of *Cranichis, C.lehmannii* Rchb. f., *C.polyantha* Schltr., and *C.pulvinifera* Garay were reported as occurring in Bolivia by Jiménez-Pérez (2011).

Recent research on *Cranichis* resulted in the description of numerous new species from the Northern Andes (e.g. Kolanowska and Szlachetko 2014; Kolanowska and Szlacheko 2019; Szlachetko and Kolanowska 2019) and in this study the diversity of this genus in Bolivia has been evaluated.

## Materials and methods

Herbaria acronyms used in this paper follow Thiers (2020). During the research on *Cranichis* over 400 specimens deposited in the herbaria: AAU, AMES, BM, C, CAY, CUVC, COL, FMB, K, LPB, MO, NY, P, PSO, RENZ, RPSC, UGDA, US, VALLE, and W were examined. Morphological characteristics of Bolivian species were prepared based exclusively on material collected in Bolivia and deposited in LPB, MO, and AMES. The morphological variation of Colombian and Ecuadorian species of *Cranichis* has been described by Szlachetko and Kolanowska (2019). Specimens examined from outside Bolivia are listed in Kolanowska and Szlachetko 2013, 2019; Szlachetko and Kolanowska 2013, 2019, and books of Szlachetko 2016; Szlachetko and Kolanowska 2020. The list of species of Bolivian *Cranichis* examined in this study is provided in Supplementary Information (Annex 1). Information on the habitats of Bolivian *Cranichis* was gathered during field studies and from the data on herbarium labels. Characteristics of species that are reported from Bolivia, but not confirmed or examined in this study, were prepared based on specimens collected in other regions, as well as the literature.

All herbarium specimens were examined in the standard way. The size and shape of the leaves and length of the scape were studied first. Then the details of the inflorescence (e.g. form of the floral bracts and ovaries) were examined. Three flowers from the middle part of the inflorescence were studied. The floral segments were observed under a stereoscopic microscope, after softening the flowers in boiling water.

Only those localities that could be identified based on information on the labels of the herbarium specimens were included in the distribution maps compiled using ArcGis 10.6 (Esri, Redlands, CA, USA).

## Nomenclature

The electronic version of this article in portable document format is a published work according to the International Code of Nomenclature for algae, fungi and plants (Turland et al. 2018) and hence the new names contained in the electronic version are effectively published under that Code. In addition, new names included in this work that were issued with identifiers by IPNI will eventually be made available to the Global Names Index. The IPNI Life Science Identifiers (LSIDs) can be obtained and the associated information viewed using any standard web browser by appending the LSID contained in this publication to the prefix http://ipni.org/. The online version of this paper is archived and available from the following digital repositories: PeerJ, PubMed Central, and CLOCKSS.

## Results

Here the characteristics of 15 Bolivian *Cranichis* are presented. The occurrence of *C.diphylla*, *C.lehmannii*, and *C.muscosa* in this country was not confirmed as we were not able to find reference material in the collection of Vasquez deposited in LPB. Moreover, in our opinion the previously published Bolivian record for *C.polyantha* is doubtful and is discussed below. Two new species of *Cranichis* are described in this paper and for the first time we are reporting the occurrence of *C.badia* and *C.longipetiolata* in Bolivia. While both Schweinfurth (1958) and Vásquez et al. (2014) accepted the name *Cranichismultiflora* (Poepp. & Endl.) Cogn., in our opinion this taxon should be classified within *Ponthieva* R.Br.

## Taxonomic treatment

### Key to Bolivian *Cranichis*

**Table:** 

1.	Margins of lateral lobes of lip irregularly erose to erose-lancinate	2
–	Lip margin entire	3
2.	Petals linear-ligulate	*C.pulvinifera*
–	Petals obliquely oblanceolate to clavate	*C.garayana*
3.	Petals glabrous	4
–	Petals ciliate, ciliate-papillate or pilose	12
4.	Lip lacks nodules on inner surface	5
–	Lip with nodules on inner surface	8
5.	Petals oblanceolate-linear	6
–	Petals linear to oblong-ligulate	7
6.	Leaf petiole up to 14 cm long, petals obliquely linear-lanceolate	*C.longipetiolata*
–	Leaf petiole up to 7.5 cm long, petals elliptical or ligulate from a short claw	*C.polyantha* (excluded)
7.	Lip base cuneate	*C.silvicola*
–	Lip base unguiculate	*C.badia*
8.	Dorsal sepal 3-veined	*C.diphylla* (not confirmed)
–	Dorsal sepal 1-veined	9
9.	Petals spathulate-obovate	*C.beckii*
–	Petals lanceolate, linear-lanceolate or linear-oblanceolate	10
10.	Ovary glandular-ciliate	*C.stictophylla* Schltr.
–	Ovary glabrous or almost so	11
11.	Inflorescence conical	*C.lehmannii* (not confirmed)
–	Inflorescence cylindrical	*C.cylindrostachys*
12.	Lip with nodules on inner surface	*C.muscosa* (not confirmed)
–	Lip lacks nodules on inner surface	13
13.	Sepals sparsely pubescent	*C.mandonii*
–	Sepals glabrous	14
14.	Lateral sepals 2- or 3-veined	*C.ciliata*
–	Lateral sepals 1-veined	15
15.	Petals ligulate-oblanceolate, ciliate along both margins	*C.atrata*
–	Petals lanceolate-ovate, obtuse, 1-veined, ciliate on the basal 2/3	*C.maldonadoana*

#### 1. Cranichis atrata

Taxon classificationPlantaeAsparagalesOrchidaceae

Schltr., Repert. Spec. Nov. Regni Veg. Beih. 7: 58. 1920.

6FE614A8-092B-5F45-A631-01B2982FFD28

##### Type.

COLOMBIA. *Madero 3* (B†; lectotype, designated by Garay (1978: 191): AMES–drawing).

##### Diagnosis.

**Figure 1.**
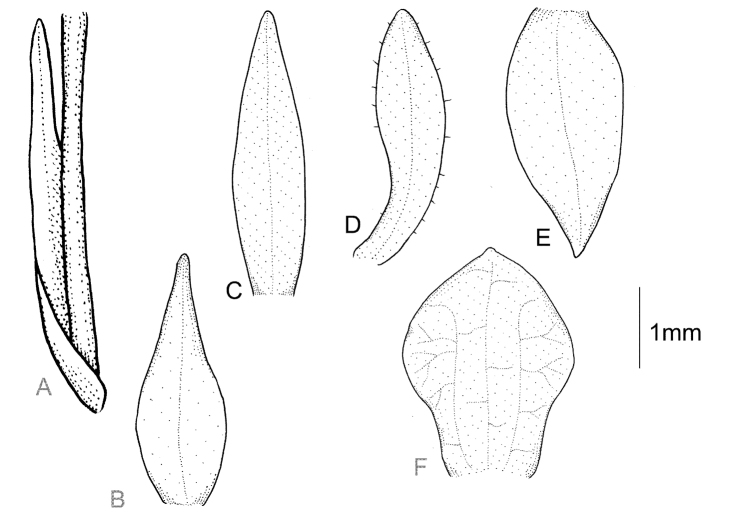
*Cranichisatrata* Schltr. **A** Ovary and floral bract. **B** Floral bract. **C** Dorsal sepal. **D** Petal. **E** Lateral sepal. **F** Lip. Drawn by P. Baranow from *R. Vasquez et al. 1429* (LPB).

Plants 24 cm tall, erect. Leaf 1, basal, petiolate; petiole 12 cm long, narrow, canaliculate; blade 9 cm long, 7 cm wide, obliquely elliptical, acuminate, base cordate. Scape glabrous, enclosed in 4 non-foliaceous sheaths. Inﬂorescence 2.5 cm long, subdensely many-ﬂowered. Flowers small, glabrous. Floral bracts 4.8 mm long, lanceolate, obtuse. Pedicellate ovary 5.0 mm long, glabrous. Dorsal sepal 3.7 mm long, 1.0 mm wide, oblong-elliptical, obtuse, 1-veined. Petals 3.1 mm long, 1.1 mm wide, obliquely ligulate-oblanceolate, obtuse, long cilia on both margins, 1-veined. Lateral sepals 3.1 mm long, 1.5 mm wide, obliquely elliptic-ovate, subacuminate, 1-veined. Lip 2.8 mm long, 2.1 mm wide, concave in the centre, subsessile, elliptic-obovate, minutely apiculate; disc with 3 thickened, dendritic branching veins. Gynostemium 1.3 mm long. Fig. 1.

##### Habitat and ecology.

Terrestrial plants growing in subhumid Tucumano-Boliviano forest at an altitude of 2200 m. Flowers in February.

##### Representative specimen.

**Figure 2.**
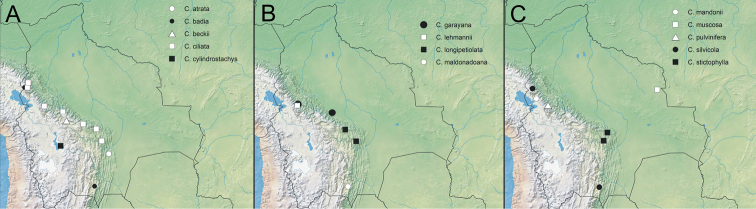
Distribution of *Cranichis* species in Bolivia. **A***C.atrata, C.badia, C.beckii, C.ciliata, C.cylindrostachys.***B***C.garayana, C.lehmannii, C.longipetiolata, C.maldonadoana*. **C***C.mandonii, C.muscosa, C.pulvinifera, C.silvicola, C. stictopylla.* Base map provided by Natural Earth (www.naturalearthdata.com).

**BOLIVIA. Santa Cruz**: Prov. Vallegrande. Río San Lorenzo, entre Piraimiri y Masicurí, 2220 m. 23 February 1991, *R. Vasquez et al. 1429* (LPB!). Fig. 2.

##### Notes.

This species is usually considered to be a synonym of *C.ciliata* (e.g. Garay 1978; Hamer 1985; Christenson 1991; Bogarín et al. 2014), however, the venation of the lateral sepals is a constant character that can be used to distinguish these two taxa. Lateral sepals of *C.atrata* are always 1-veined (vs. 2- or 3-veined).

#### 2. Cranichis badia

Taxon classificationPlantaeAsparagalesOrchidaceae

Renz ex Kolan. & Szlach., Nordic J. Bot. 32(3): 289. 2014.

0EEE3076-6E1F-545C-9353-796A5137DB10

##### Type.

VENEZUELA. *Renz 6065* (holotype: RENZ!; isotypes: RENZ!).

##### Diagnosis.

**Figure 3.**
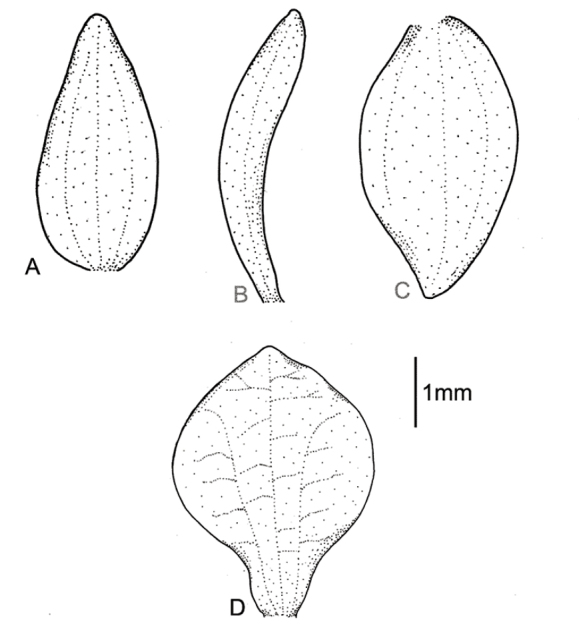
*Cranichisbadia* Renz *ex* Kolan. & Szlach. **A** Dorsal sepal. **B** Petal. **C** Lateral sepal. **D** Lip. Drawn by P. Baranow from *L. Cayola et al. 3657* (LPB).

Plants 28–60 cm tall. Leaf 1, basal, petiolate; petiole 10–19 cm long, narrow, canaliculate; blade 7–11 cm long, 5 cm wide, ovate-elliptical, acuminate, cuneate at the base. Scape sparsely pubescent on upper part, enclosed in 4-5, non-foliaceous sheaths. Inﬂorescence 6–8 cm long, cylindrical, subdensely many-ﬂowered. Flowers brownish or greenish-yellow. Floral bracts 4.0–4.5 mm long, lanceolate, acuminate, glabrous. Pedicellate ovary 5.3–6.2 mm long, glabrous. Dorsal sepal 3.3–4.0 mm long, 1.5 mm wide, ovate, obtuse, 3-veined. Petals 3.7–4 mm long, 0.6–0.7 mm wide, falcately linear to linear-lanceolate, apex rounded, 1-veined, glabrous. Lateral sepals 4.0–4.1 mm long, 1.5–2.1 mm wide, obliquely elliptical, obtuse, 2- or 5-veined. Lip 2.9–3.6 mm long, 2.3–2.6 mm wide, concave, unguiculate, elliptic-suborbicular above, obtuse; disc 3-veined, midvein anastomosing, lateral veins branching. Gynostemium 1.5–2 mm long. Fig. 3.

##### Habitat and ecology.

Terrestrial in Yungas montane humid forest with *Weinmannia* L. (Cunoniaceae), *Clethra* L. (Clethraceae), *Ocoteamandonii* Mez (Lauraceae), numerous plants of *Chusquea* Kunth (Poaceae) and *Piper* L. (Piperaceae). It occurs at altitudes between 2150-2890 m. Flowers in March and May.

##### Representative specimens.

**BOLIVIA. La Paz**: Prov. B. Saavedra. Area Natural de Manejo Integrado Apolobamba, Wayrapata. 15°06’45”S 68°55’05”W, 2889 m. 8 May 2010. *L. Cayola et al. 3657* (LPB!). **Tarija**: Prov. Cercado, cerca Victoria, 2150 m. 3 March 1986. *E. Bastian 937* (LPB!). Fig. 2.

##### Notes.

Bolivian plants are somewhat different from typical plants of *C.badia.* Lateral sepals of *Cayola et al. 3657* are 2-veined and lateral sepals of *Bastian 937* are 5-veined, whereas typically *C.badia* has 3 veins. *Cranichisbadia* was described relatively recently (Kolanowska and Szlachetko 2014) and its morphological variation requires further study.

#### 3. Cranichis beckii

Taxon classificationPlantaeAsparagalesOrchidaceae

Kolan., Baranow, S. Nowak & A. Fuentes, sp. nov.

63E0620A-F4BA-5092-A46F-568E99E8FD76

urn:lsid:ipni.org:names:77233921-1

##### Type.

BOLIVIA. *Beck 313* (holotype: LPB!).

##### Diagnosis.

Species similar to *C.lehmannii*, but distinguished by larger leaves up to 20 cm long, 1-veined lateral sepals, spathulate-obovate petals and ciliate ovary.

**Figure 4.**
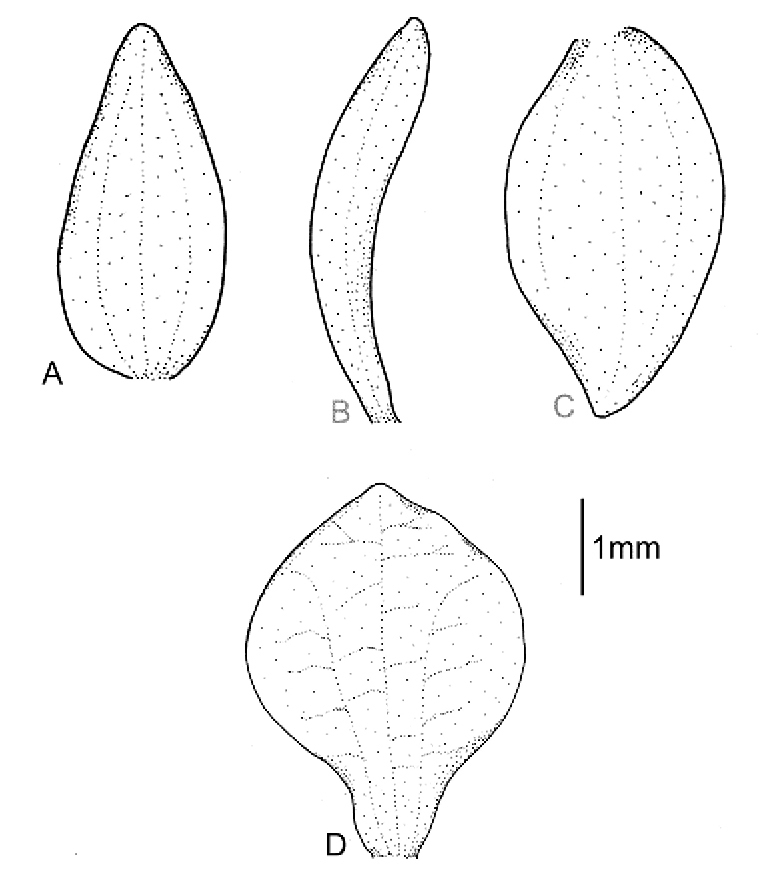
*Cranichisbeckii* sp. nov. **A** Ovary and gynostemium. **B** Floral bract. **C** Dorsal sepal. **D** Petal. **E** Lateral sepal. **F** Lip, front view. **G** Lip, side view. Drawn by P. Baranow from *S. G. Beck 313 et al.* (LPB).

Plants 50 cm tall. Leaves 3, basal, petiolate; petiole 8–10 cm long, narrow; blade 11–20 cm long, 4–6 cm wide, ovate, acute. Scape erect, enclosed in about 6, foliaceous sheaths. Inﬂorescence 16 cm long, conical, sublaxly many-ﬂowered. Flowers yellowish, glabrous. Floral bracts 6.2 mm long, lanceolate, acute, microscopically ciliate. Pedicellate ovary 9.5 mm long, microscopically ciliate. Dorsal sepal 3.5 mm long, 1.5 mm wide, oblong-ovate, acuminate, obtuse, 1-veined. Petals 3.2 mm long, 1.3 mm wide, obliquely spathulate-obovate, apiculate, 1-veined. Lateral sepals 3.5 mm long, 1.7 mm wide, obliquely oblong-elliptical, subapiculate, concave near base, 1-veined. Lip 3 mm long, 2.1 mm wide, concave, subsessile, elliptical to oblong-elliptical in outline, apiculate at apex; disc deeply concave in the centre with numerous, irregularly subglobose thickenings on inner surface, veins 3, thickened. Gynostemium 2 mm long. Fig. 4.

##### Etymology.

Dedicated to Stephan G. Beck, who collected the type specimen and is a former director of Herbario Nacional de Bolivia.

##### Habitat and ecology.

Terrestrial in Yungas humid, secondary montane forest at an altitude of ca. 1730 m. Flowers in February.

##### Representative specimen.

**BOLIVIA. La Paz**: Prov. Nor Yungas. Cotapata. Estación Biológica de Tunkini. A media hora de la EBT, cruzando l río, 1735 m. 2 February 2002. *S. G. Beck 313* (LPB!). Fig. 2A, 4.

##### Notes.

This species resembles *C.lehmannii* in general flower morphology, but its petals are spathulate-obovate, widest apically and similar in shape to those of *C.diphylla*. Leaves of *C.lehmannii* are smaller (up to 11 cm long), its ovary is glabrous (microscopically ciliate in *C.beckii*), lateral sepals are 2-veined (vs 1-veined in *C.beckii*) and petals are lanceolate. The comparative morphology of *C.beckii* and *C.lehmannii* is presented in Table 1.

**Table 1.** Comparative morphology of *C.beckii* and *C.lehmannii.*

Character	*C.beckii*	*C.lehmannii*
Leaves	3, petiole 8–10 cm long; blade 11–20 x 4–6 cm, ovate, acute	1–3, petiole 3–4(8) cm long; blade 6.5–11 x 2.8–5 cm, ovate, acute
Inflorescence	16 cm, conical, sublaxly many-ﬂowered	2.5–10.5 cm long, conical, sublaxly many-ﬂowered
Ovary	9.5 mm long, microscopically ciliate	6-9 mm long, almost glabrous
Floral bracts	6.2 mm long, lanceolate, acute, microscopically ciliate	4.5–8 mm long, lanceolate, acute, glabrous
Dorsal sepal	3.5 x 1.5 mm, oblong-ovate, acuminate, obtuse, 1-veined	3–4 mm x 1–1.1 mm, oblong-lanceolate to oblong ovate, acuminate, obtuse, concave, 1-veined
Lateral sepals	3.5 x 1.7 mm, obliquely oblong-elliptic, subapiculate, concave near the base, 1-veined	3.5–4 x 1.5–1.7 mm, obliquely elliptic-ovate to elliptic-lanceolate, subacute to subapiculate, concave in the center, obscurely 2-veined
Petals	3.2 x 1.3 mm, obliquely spathulate-obovate, apiculate, 1-veined	2.5–3.5 x 0.5–1.2 mm, lanceolate, somewhat oblique at base, subobtuse, 1-veined
Lip shape	3 x 2.1 mm, elliptic to oblong-elliptic in outline, apiculate at apex	3–3.3 x 1.6–2.3 mm, elliptic to oblong-elliptic in outline, obtuse at apex, lateral margins reﬂexed
Lip disc	with numerous, irregularly subglobose thickenings on the inner surface, veins thickened	with numerous, irregularly subglobose thickenings on the inner surface, veins thickened

#### 4. Cranichis ciliata

Taxon classificationPlantaeAsparagalesOrchidaceae

(Kunth) Kunth, Syn. Pl. 1: 324. 1822.

18E7EA7D-FF2E-50B8-A6AB-E441AAB758D8

Ophrys ciliata Kunth, Nov. Gen. Sp. (quarto ed.) 1: 334, t. 74. 1816.

##### Type.

VENEZUELA. *Humboldt s.n*. (lectotype, designated by Garay (1978: 191): W!).

##### Diagnosis.

**Figure 5.**
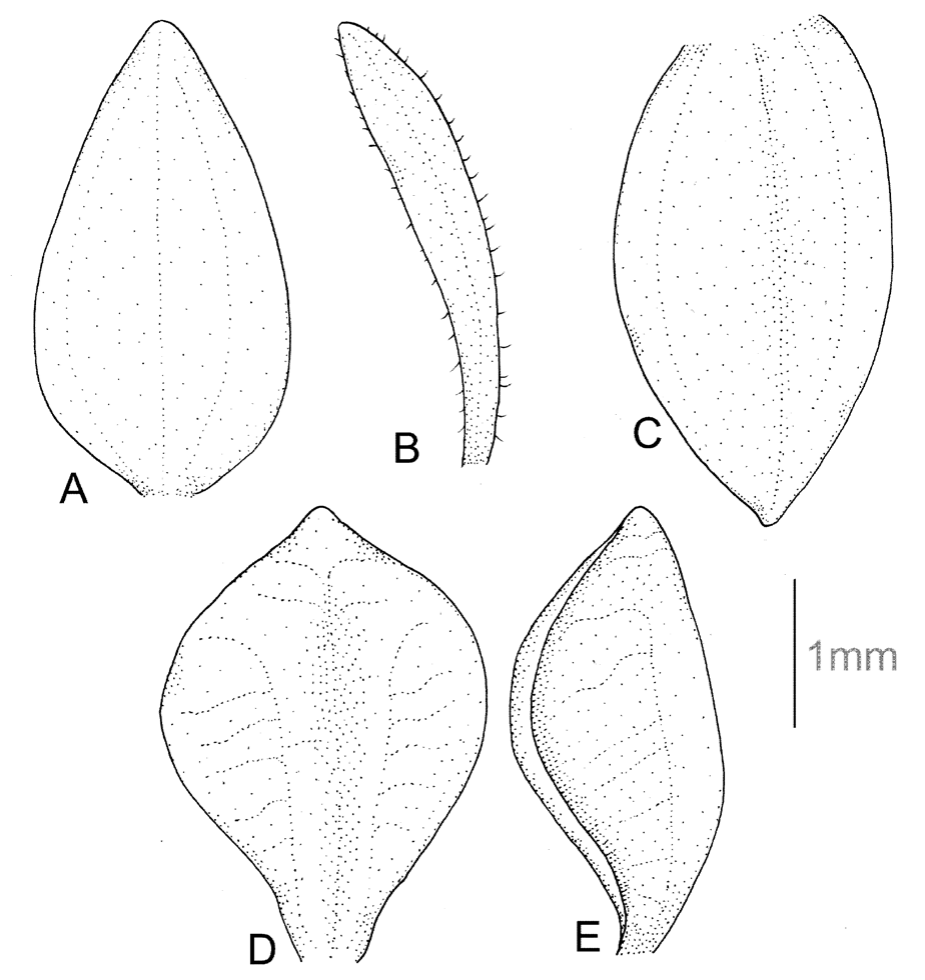
*Cranichisciliata* (Kunth) Kunth. **A** Dorsal sepal. **B** Petal. **C** Lateral sepal. **D** Lip, front view. **E** Lip, side view. Drawn by P. Baranow from *I. Loza et al. 1621A* (LPB).

**Figure 6.**
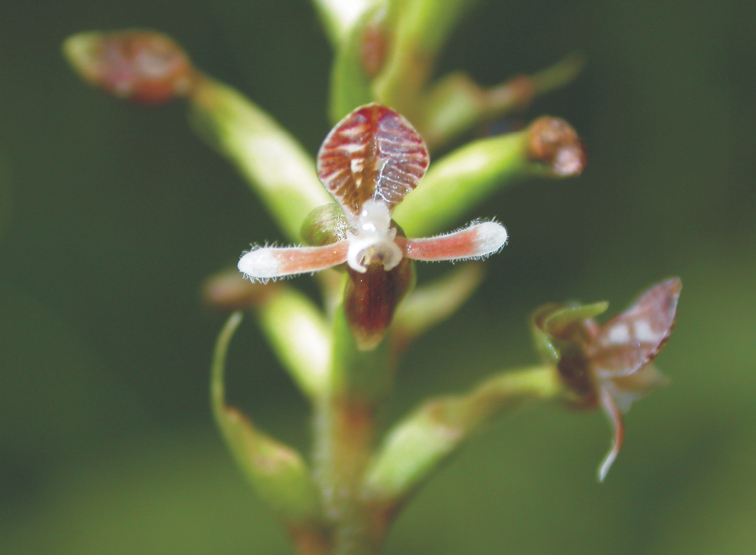
*Cranichisciliata* (Kunth) Kunth (photo by A. Fuentes).

Plants 26–54 cm tall. Leaves l–2, basal, petiolate; petiole 5–19 cm long, canaliculate; blade 4–15 cm long, 3–7.5 cm wide, oblong-ovate to elliptical, acute to acuminate, broadly rounded to subcordate at the base. Scape glabrous in lower part, glandular-pubescent above, enclosed in 3–6 non-foliaceous sheaths. Inﬂorescence 3.5–17 cm long, cylindrical, sublaxly to subdensely many-ﬂowered. Flowers whitish marked with green or purple-brown, with reddish or brown lip. Floral bracts 4.2–6.0 mm long, ovate-lanceolate, ovate, acuminate to acute, glabrous. Pedicellate ovary 5.0–8.5 mm long, glabrous. Dorsal sepal 3.0–4.1 mm long, 1.2–2.0 mm wide, oblong-elliptical to ovate, obtuse, 3-5-veined (rarely 1-veined). Petals 2.8–4.1 mm long, 0.4–1.0 mm wide, obliquely narrowly-ligulate to oblanceolate, obtuse, 1-veined, margins ciliate. Lateral sepals 3.0–4.5 mm long, 1.2–2.0 mm wide, obliquely oblong-ovate to elliptic-ovate, subobtuse, 2- or 3-veined. Lip 2.5–3.2 mm long, 2.1–3.0 mm wide, gibbose at base, cochleate above, obovate to suborbicular above the base, rounded or obtuse at apex; disc with 3 thickened, dendritic branching veins. Gynostemium 1.2–2 mm long. Fig. 5–6.

##### Habitat and ecology.

Terrestrial in Yungas secondary submontane humid forest, montane, cloud forest, secondary forest with *Tibouchina* Aubl. (Melastomataceae) and *Miconia* Ruiz & Pav. (Melastomataceae), forest with *Clusia* L. (Clusiaceae) and *Weinmannia* L. (Cunoniaceae) and in Tucumano-Boliviano secondary submontane humid forest with *Myrcianthes* O. Berg. (Myrtaceae). Flowers in March, April, May and June. The populations of this species were recorded growing at altitudes between 1900-3000 m. According to Vásquez et al. (2014) this species occurs in the Yungas ecoregion at altitudes between 1000–3000 m.

##### Representative specimens.

**BOLIVIA. Cochabamba**: Cochabamba: Prov. Carrasco. La Siberia. January 1983. *R. Vásquez et al. 792* (Herbarium Vasquezianum–Dodson and Vásquez. 1989); Sehuencas, PN Carrasco, 2100 m. 5 April 1996. *P. Ibish & C. Ibish 96.0020* (LPB!), Prov. Ayopaya, 10 km Cocapata-Cotacajes, 3000 m. 9 May 1997. *M. Kessler et al. 9412* (LPB!). **La Paz**: Prov. Nor Yungas. 2.4 km below Chuspipata on road to Chulumani, 2950 m. 4 March 1983. *J. C. Solomon 9678* (LPB!). Prov. B. Saavedra. Area Natural de Manejo Integrado Apolobamba. Chulina, al frente de estancia Wikjelani, pasando por el río Sillaca. 15°07’57”S 68°52’57”W, 2760 m. 17 April 2010. *A. Fuentes & R. Rodas 16115* (LPB!), Area Natural de Manejo Integrado Apolobamba. Kazu, cruzando el río Sillaca, 30 minutos bajando por el río Sillaca, al frente de la loma Wakelli. 15°07’53”S 68°53’08”W, 2788 m. 17 April 2010. *I. Loza et al. 1621A* (LPB!), Area Natural de Manejo Integrado Apolobamba. Paian, río Silliaca, sector Kumamita. 15°06’47”S 68°55’04”W, 2659 m. 20 April 2010. *I. Loza et al. 1698* (LPB!), Prov. Franz Tamayo. Parque Nacional Madidi. Sector campamento Tanhuara, por el antiguo camino Pelechuco-Apolo, pasando el río Pelechuco. 14°44’59”S 68°56’57”W, 1905 m. 28 June 2009. *A. Fuentes & D. Alanes 15018* (LPB!). **Santa Cruz**: Prov. Vallegrande. Vallegrande, aprox. 10 km al S por el camino a Pucará, 2260 m. 31 March 2001. *A. Fuentes 2903* (LPB!). Fig. 2.

##### Notes.

This is a widely distributed species reported from Mexico, Guatemala, El Salvador, Honduras, Nicaragua, Costa Rica, Panama, Colombia, Venezuela, Ecuador, Peru, Bolivia and Argentina. Various authors have synonymized *C.ciliata* with different species, e.g. *C.antioquiensis* Schltr. (Davidse et al. 2020), *C.atrata* (Garay 1978; Christenson 1991; Bogarín et al. 2014), *C.curtii* Schltr. (Davidse et al. 2020), *C.irazuensis* Schltr. (Davidse et al. 2020), *C.mandonii* (Schweinfurth 1958; Bogarín et al. 2014), *C.pachnodes* Løjtnant (Bogarín et al. 2014), *C.pleioneura* (Bogarín et al. 2014), *C.polyblephara* Schltr. (Brako and Zarucchi 1993; Bogarín et al. 2014), *C.schlimii* Rchb. f. (Bogarín et al. 2014) and *C.sororia* Schltr. (Garay 1978; Bogarín et al. 2014). The actual taxonomic position of most of them requires further study.

#### 5. Cranichis cylindrostachys

Taxon classificationPlantaeAsparagalesOrchidaceae

Schltr., Repert. Spec. Nov. Regni Veg. Beih. 7: 59. 1920.

C12C12C0-AE78-5ADF-B8B7-0A3A7AFEF898

##### Type.

COLOMBIA. *Madero 14* (B†, lectotype, designated by Garay (1978: 199): AMES!–drawing).

##### Diagnosis.

**Figure 7.**
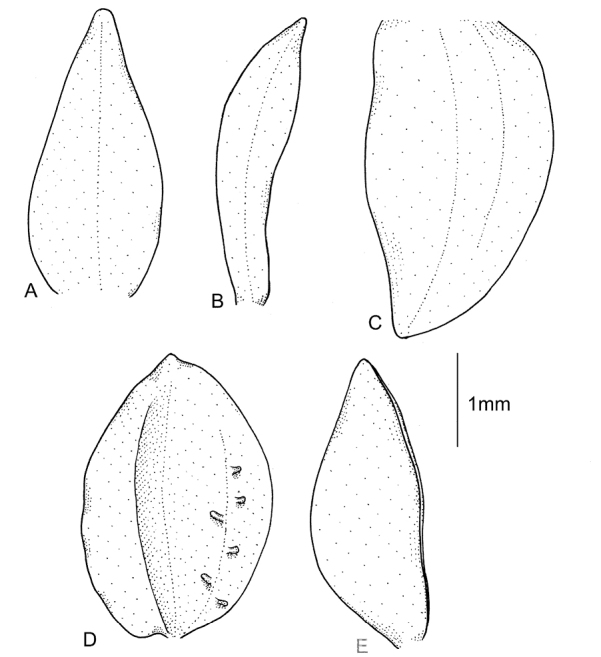
*Cranichiscylindrostachys* Schltr. **A** Dorsal sepal. **B** Petal. **C** Lateral sepal. **D** Lip, front view. **E** Lip, side view. Drawn by P. Baranow from *F. Miranda 1236 et al.* (LPB).

Plants 26–29 cm tall. Leaves 2–3, basal, petiolate; petiole 3–8 cm long, narrow, canaliculate; blade 2–6 cm long, 1.6–4 cm wide, ovate, acute, base obliquely cordate to cuneate. Scape glabrous, remotely 4–5-sheathed. Inﬂorescence 5–12 cm long, cylindrical, subdensely many-ﬂowered. Flowers small, glabrous. Floral bracts 3.8 mm long, ovate-lanceolate, acuminate, glabrous. Pedicellate ovary 5.5 mm long, glabrous. Dorsal sepal 3 mm long, 1.3 mm wide, ovate-lanceolate, subobtuse, 1-veined. Petals 3.1 mm long, 0.7 mm wide, obliquely lanceolate to linear-oblanceolate, subobtuse, glabrous on margins, 1-veined. Lateral sepals 3.6 mm long, 2.1 mm wide, obliquely elliptic-ovate, slightly concave at base, obtuse, 2-veined. Lip 3.1 mm long, 2 mm wide, somewhat concave, subsessile, oblong-elliptical, shortly apiculate; disc with 3 thickened, dendritic branching veins with prominent nodules. Gynostemium 1.8 mm long. Fig. 7.

##### Habitat and ecology.

Terrestrial in Yungas montane secondary forest at an altitude of 1980 m. Flowers in March.

##### Representative specimen.

**BOLIVIA. La Paz**: Prov. Yungas, Challapata, pasando ladera quemada más alla de las Masdevallias, 1981 m. 4 March 2006. *F. Miranda et al. 1236* (LPB!). Fig. 2.

##### Notes.

*Cranichiscylindrostachys* is often considered to be a synonym of *C.lehmannii* (e.g. Garay, 1978). The two species differ in leaf petiole length (usually 3-4 cm long in *C.lehmannii*) and inflorescence architecture (conical in *C.lehmannii*), but whether they are different species is doubtful and further molecular studies are needed to clarify the situation.

#### 6. Cranichis diphylla

Taxon classificationPlantaeAsparagalesOrchidaceae

Sw., Prodr. 120. 1788.

334B38A6-2066-5393-8C8B-BD49CFBA954E

##### Type.

JAMAICA. *s.n.* (lectotype, designated by Garay (1978: 192): BM!; isolectotypes, LD, S!, UPS, W!; AMES! -drawing).

##### Diagnosis.

**Figure 8.**
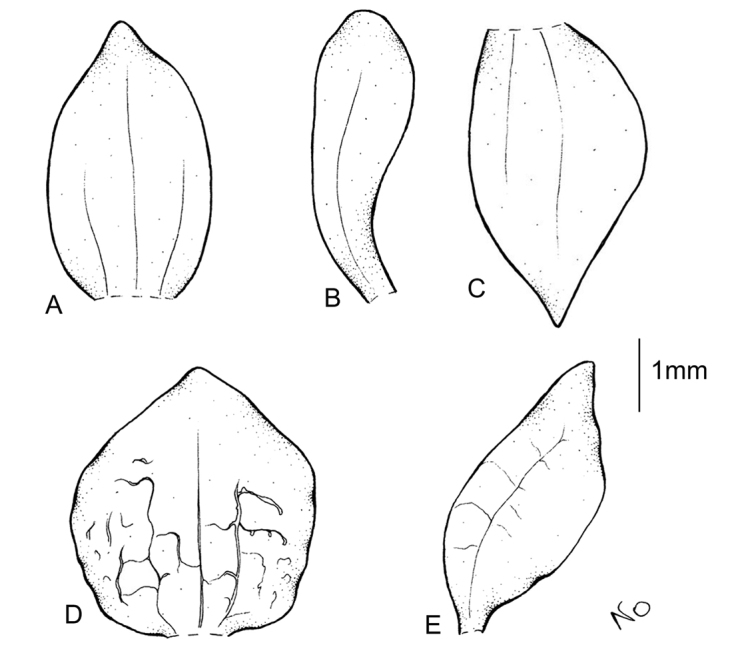
*Cranichisdiphylla* Sw. **A** Dorsal sepal. **B** Petal. **C** Lateral sepal. **D** Lip, front view. **E** Lip, side view. Drawn by N. Olędrzyńska from *Killip & Smith 15946* (AMES).

Plants up to 40 cm tall. Leaves 1–3, basal, often variegated, petiolate; petiole rather variable in length, up to 3 cm; blade up to 9 cm long, 4 cm wide, ovate to ovate-lanceolate, acute to subacuminate, subcordate at base. Scape slender, erect, remotely few-sheathed, glabrous below, glandular-pubescent above. Inﬂorescence up to 6.5 cm long, cylindrical, loosely to subdensely many-ﬂowered. Flowers white with green veins. Floral bracts 4 mm long, ovate-lanceolate, acuminate, sparsely glandular. Pedicellate ovary up to 6 mm long, cylindrical, more or less glandular. Dorsal sepal up to 3.5 mm long, 1.6 mm wide, erect, elliptical, subacute to subobtuse, 3-veined, occasionally sparsely pubescent dorsally. Petals up to 3.1 mm long, 1 mm wide, near apex linear-oblanceolate, acute to obtuse, 1-veined, glabrous along margins. Lateral sepals up to 4 mm long, 1.6 mm wide, spreading, obliquely ovate to ovate-elliptical, acute to obtuse, 2-veined, occasionally sparsely pubescent dorsally. Lip up to 3.6 mm long, 3.2 mm wide, concave, inconspicuously subcordate at base, ovate to broadly elliptical in outline, subacute to subobtuse; disc obcordately papillose-thickened with three branching, often glandular (with nodules) veins from base to middle of lip. Gynostemium 1.3–2 mm long. Fig. 8.

##### Habitat and ecology.

According to Vásquez et al. (2014) this species occurs in the Yungas ecoregion at altitudes between 1000–1500 and 2500–3500 m.

##### Representative specimen.

**BOLIVIA. Santa Cruz**. *R. Vásquez C. 2180* (herb. Vásquez, LPB).

##### Notes.

The specimen cited by Vásquez et al. (2014) was not found in the Orchid collection in LPB and we were not able to confirm the occurrence of this species in Bolivia. The characteristics presented above were prepared based on those of Ecuadorian and Colombian plants (Szlachetko and Kolanowska 2019).

#### 7. Cranichis garayana

Taxon classificationPlantaeAsparagalesOrchidaceae

Dodson & R. Vásquez, Icon. Pl. Trop., ser. 2. 3: pl. 210. 1989.

2EEC19E7-543A-5A65-B3FE-48E4CC317558

##### Type.

BOLIVIA. *Vásquez Ch. 612* (holotype MO).

##### Diagnosis.

**Figure 9.**
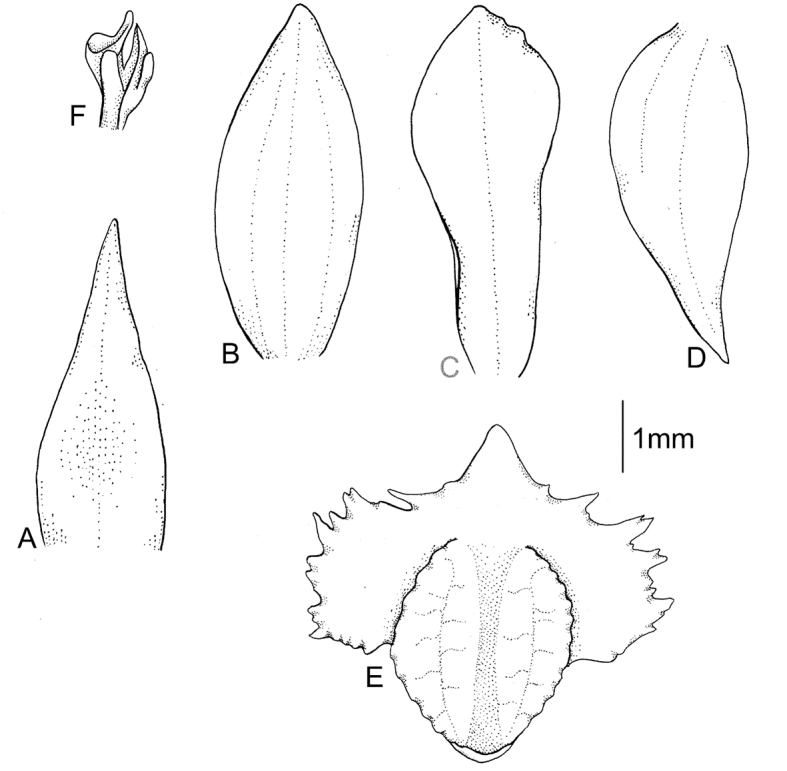
*Cranichisgarayana* Dodson & R. Vásquez. **A** Floral bract. **B** Dorsal sepal. **C** Petal. **D** Lateral sepal. **E** Lip. **F** Gynostemium. Drawn by P. Baranow from *I. Jimenez 3854 & Miranda F.* (LPB).

Plants 20–40 cm tall. Leaves 2, basal, petiolate; petiole 4–6 cm long, narrow, canaliculate; blade 2.8–8.0 cm long, 3.0–5 cm wide, ovate, acuminate. Scape glabrous, enclosed in about 4–5 non-foliaceous sheaths. Inﬂorescence 4.0–10 cm long, cylindrical, sub-laxly few- to many-ﬂowered. Flowers whitish-brown, glabrous. Floral bracts 5.0 mm long, lanceolate, acute. Pedicellate ovary 7.5 mm long, almost glabrous. Dorsal sepal 5.0–6.0 mm long, 2.3–3.0 mm wide, oblong-elliptical to ovate, obtuse, 3-veined. Petals 4.5–5.0 mm long, 1.7–2.0 mm wide, obliquely oblanceolate to spathulate, obtuse, 1-veined, glabrous. Lateral sepals 5.1–6.0 mm long, 2.3–3.5 mm wide, obliquely ovate, obscurely 2-veined. Lip 4.5–5.0 mm long, 4.5–5.0 mm wide, concave, subsessile, 3-lobed above the elliptical base, lateral lobes subquadrate, deeply laciniate, middle lobe ovate, rounded or obtuse; disc with 5 dendritic branching veins. Gynostemium 2.9 mm long. Fig. 9.

##### Habitat and ecology.

Terrestrial or epiphytic in Yungas montane wet forest at altitudes between 1880-2240 m. Flowers in March. According to Vásquez et al. (2014) this species occurs in Yungas ecoregion at altitudes between 1500–2500 m.

##### Representative specimens.

**BOLIVIA. Cochabamba**: Chapare, km 100, Cochabamba to Villa Tunari, 1880 m. 22 March 1981. *R. Vásquez Ch. 612* (MO). **La Paz**: Prov. Nor Yungas. PN-ANMI Cotapata, sendero Sandillani al segundo campamento, 2240 m. 4 March 2006. *I. Jimenez & F. Miranda 3854* (LPB!). Fig. 2.

##### Notes.

*Cranichisgarayana* is a Bolivian endemic easily distinguished from other species by its 3-lobed lip above an elliptical base with subquadrate, deeply laciniate, lateral lobes and ovate, rounded middle lobe.

#### 8. Cranichis lehmannii

Taxon classificationPlantaeAsparagalesOrchidaceae

Rchb. f., Otia Bot. Hamburg. 1: 4. 1878.

95D624BD-0285-5997-839F-1E3EFD3CA3D0

##### Type.

ECUADOR. *Lehmann 77* (lectotype, designated by Garay (1978: 199): W!; AMES!–drawing, UGDA-DLSz!–drawing).

##### Diagnosis.

**Figure 10.**
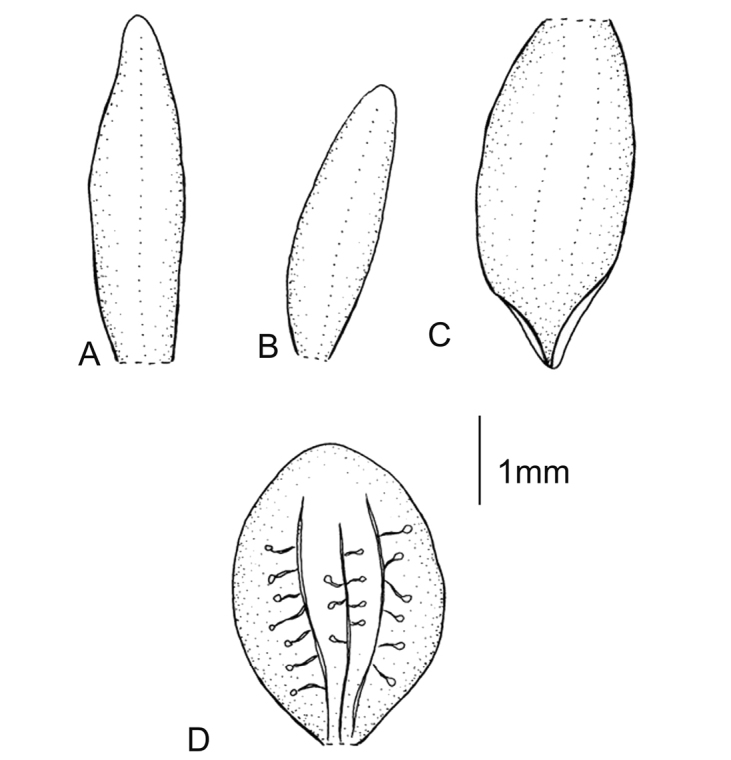
*Cranichislehmannii* Rchb. f. **A** Dorsal sepal. **B** Petal. **C** Lateral sepal. **D** Lip. Redrawn by A. Król from Garay’s illustration of specimen collected by *Lehmann 77* (W).

Plants 26–60 cm tall. Leaves 1–3, basal, petiolate; petiole 3–4(8) cm long, narrow, canaliculate; blade 6.5–11 cm long, 2.8–5 cm wide, ovate, acute. Scape erect, enclosed in 6–9 sheaths. Inﬂorescence 2.5–10.5 cm long, conical, sublaxly many-ﬂowered. Flowers small, glabrous. Floral bracts 4.5–8 mm long, lanceolate, acute. Pedicellate ovary 6-9 mm long, almost glabrous. Dorsal sepal 3–4 mm long, 1–1.1 mm wide, oblong-lanceolate to oblong ovate, acuminate, obtuse, concave, 1-veined. Petals 2.5–3.5 mm long, 0.5–1.2 mm wide, lanceolate, somewhat oblique at base, subobtuse, 1-veined. Lateral sepals 3.5–4 mm long, 1.5–1.7 mm wide, obliquely elliptic-ovate to elliptic-lanceolate, subacute to subapiculate, concave in the centre, obscurely 2-veined. Lip 3–3.3 mm long, 1.6–2.3 mm wide, concave, subsessile, elliptical to oblong-elliptical in outline, obtuse at apex, lateral margins reﬂexed; disc with numerous, irregularly subglobose thickenings on the inner surface, veins thickened with dendritic branching. Gynostemium 1.2–1.5 mm long. Fig. 10.

##### Habitat and ecology.

According to Vásquez et al. (2014) this species grows as an epiphyte in the Yungas ecoregion at altitudes between 2500–3500 m.

##### Representative specimen.

**BOLIVIA. La Paz**: PN-ANMI Cotapata, sendero Chojllapata, poco antes de llegar al codo del sendero. 16°14’S, 67°52’O, 2670 m. *I. Jiménez 5579* (LPB–Jiménez-Pérez 2011). Fig. 2.

##### Notes.

The specimen cited by Jiménez-Pérez (2011) was not found in the Orchid collection deposited in LPB and we were not able to confirm the occurrence of this species in Bolivia. The characteristics presented above are based on those of Ecuadorian and Colombian plants (Szlachetko and Kolanowska 2019).

#### 9. Cranichis longipetiolata

Taxon classificationPlantaeAsparagalesOrchidaceae

C. Schweinf., Amer. Orchid Soc. Bull. 21: 268. 1952.

63AC2BFE-E73E-5D90-B33D-C3E45A866F34

##### Type.

PERU. *Ferreyra 3120* (lectotype, designated by Garay (1978: 200): AMES!, isolectotype: USM; UGDA-DLSz!–drawing).

##### Diagnosis.

**Figure 11.**
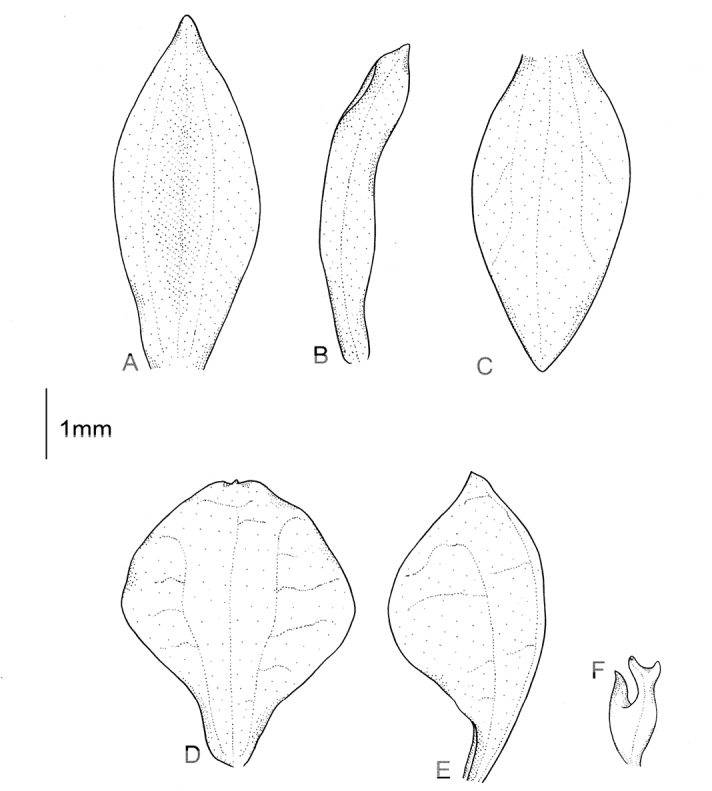
*Cranichislongipetiolata* C. Schweinf. **A** Dorsal sepal. **B** Petal. **C** Lateral sepal. **D** Lip, front view. **E** Lip, side view. **F** Gynostemium. Drawn by P. Baranow from *M. Mendoza & S. Acebo 912* (LPB).

Plants up to 29-37 cm tall. Leaf 1, basal, petiolate; petiole 10.5–14 cm long; blade 8–10 cm long, ca. 5.0 cm wide, oblong-elliptical to ovate, oblique, acute or shortly acuminate, cuneate to subcordate at base. Scape glabrous below, ﬁnely pubescent or ciliate above, with 3–5, non-foliaceous sheaths. Inﬂorescence 5–7 cm long, conical, subdensely many-ﬂowered. Flowers greenish or greenish-white. Floral bracts 4.3 mm long, lanceolate, glabrous. Pedicellate ovary 6.2 mm long, glandular. Dorsal sepal 4.1–5.0 mm long, 1.2–2.1 mm wide, ovate-lanceolate to narrowly elliptical, subacute, concave, 3-veined. Petals 3.3–4.0 mm long, 0.3–0.7 mm wide, obliquely linear-lanceolate, subobtuse, more or less oblique or curved, 1-veined, glabrous. Lateral sepals 4.2 mm long, 1.6–2 mm wide, obliquely elliptical, subacute to obtuse, 2–3-veined, margin very sparsely ciliate. Lip 3.2–3.6 mm long, 1.8–3.0 mm wide, deeply concave, basally gibbose, shortly unguiculate, obovate to suborbicular, apex rounded with a minute apiculus; disc with three transversely anastomosing veins. Gynostemium 1.8–2.0 mm long. Fig. 11.

##### Habitat and ecology.

Terrestrial in Yungas montane humid, and cloud forest, also in montane Tucumano-Boliviano forest, at altitudes between 2380-3000 m. Flowers in March.

##### Representative specimens.

Bolivia. Santa Cruz: J.M. Caballero, Comunidad Siberia, ca. 1-2 km arriba del pueblo de Siberia, sobre un camino vecinal, entrando hacia el Parque Nacional Amboro. 17°49.36’S, 64°45.14’W, 3001 m. 26 March 2004. *M. Mendoza & S. Acebo 912* (LPB!), Vallegrande. Tucumano-Bolivano. 18°34’28”S 64°02’33”W, 2387 m. *Parada et al. 4208* (LPB!). Fig. 2.

##### Notes.

The lip of the specimen collected by *Parada et al. 4208* (LPB) differs somewhat in shape from the typical form of that of *C.longipetiolata*, which is obovate rather than suborbicular in outline.

#### 10. Cranichis maldonadoana

Taxon classificationPlantaeAsparagalesOrchidaceae

Kolan., Baranow, S. Nowak & A. Fuentes, sp. nov.

343BE080-A8C4-57F4-AA81-86CC87F99845

urn:lsid:ipni.org:names:77233922-1

##### Type.

BOLIVIA. *Bastian 937* (holotype LPB!; isotype LPB!).

##### Diagnosis.

Species similar to *C.pleioneura*, but distinguished by 1-veined sepals, lanceolate-ovate petals that are sparsely ciliate in the lower part and by subsessile, suborbicular-obovate lip with apiculate apex.

**Figure 12.**
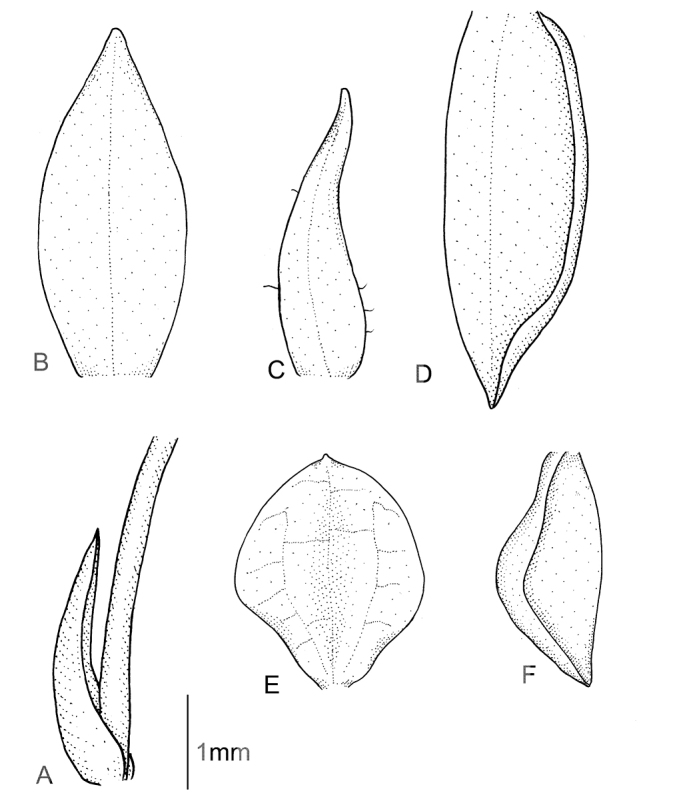
*Cranichismaldonadoana* sp. nov. **A** Ovary and floral bract. **B** Dorsal sepal. **C** Petal. **D** Lateral sepal. **E** Lip, front view. **F** Lip, side view. Drawn by P. Baranow from *E. Bastión 937* (LPB).

Plants 30–40 cm tall, erect. Leaf 1, basal, petiolate; petiole 11–14 cm long, narrow, canaliculate; blade 7.8–10 cm long, 4.2–6 cm wide, ovate, apex not preserved. Scape enclosed in 4–5 sheaths, glandular towards apex. Inﬂorescence 6–7 cm long, cylindrical, densely many-ﬂowered. Flowers greenish-yellow, glabrous. Floral bracts 3.3 mm long, lanceolate, acuminate, glabrous. Pedicellate ovary 4.2 mm long, glabrous. Dorsal sepal 3.7 mm long, 1.5 mm wide, oblong-elliptical, obtuse, 1-veined. Petals 3.2 mm long, 1 mm wide, obliquely lanceolate-ovate, obtuse, 1-veined, ciliate on basal 2/3. Lateral sepals 4.2 mm long, 1.3 mm wide, obliquely elliptic-ovate, subacuminate, subobtuse, 1-veined. Lip 2.4 mm long, 2 mm wide, lower part concave, subsessile, suborbicular-obovate, apex apiculate; disc with 3 thickened, dendritic branching veins. Gynostemium 2.3 mm long. Fig. 12.

##### Etymology.

Dedicated to Carla Maldonado, the director of Herbario Nacional de Bolivia, for her great support during our studies in Bolivia.

##### Habitat and ecology.

Terrestrial plants growing in Tucumano-Boliviano pine (Podocarpaceae) forest at an altitude of 2100 m. Flowers in March.

##### Representative specimen.

**BOLIVIA. Tarija**: Prov. Cercado, cerca Victoria, 2150 m. 3 March 1986. *E. Bastian 937* (LPB!). Fig. 2B, 12.

##### Notes.

This species resembles *C.pleioneura* and *C.barkleyi* Szlach. & Kolan., from which it differs in its lanceolate-ovate petals (vs. linear-oblanceolate to linear-ligulate), which are rather sparsely ciliate on the lower part (vs. equally ciliate or pilose along whole length). Unlike in *C.pleioneura* those of *C.maldonadoana* are 1-veined. *Cranichisatrata* Schltr. differs from *C.maldonadoana* by having ligulate-oblanceolate petals, which are ciliate along both margins (vs. lanceolate-ovate, obtuse, 1-veined, ciliate on the basal 2/3). The comparative morphology of *C.maldonadoana*, *C.barkleyi* and *C.pleioneura* is presented in Table 2.

**Table 2.** Comparative morphology of *C.maldonadoana, C.barkleyi* and *C.pleioneura*.

Character	*C.maldonadoana*	*C.barkleyi*	*C.pleioneura*
Leaves	1, petiole 11–14 cm long; blade 7.8–10 x 4.2–6 cm, ovate, apex not preserved	1, petiole 9–17 cm long; blade 9–10 x 4–7 cm, ovate, ovate-elliptic, acute or acuminate	1, petiole 8–15 cm long; blade 7.5–15 x 3.5–7.5 cm, oblong-ovate, acute to acuminate
Inflorescence	6–7 cm long, cylindric, densely many-ﬂowered	3–4 cm long, cylindric, loosely many-flowered	5–12 cm long, cylindric, densely many-flowered
Ovary	4.2 mm long, glabrous	8–9 mm long, glabrous	5–9 mm long, glabrous
Floral bracts	3.3 mm long, lanceolate, acuminate, glabrous	5–8 mm long, lanceolate, acuminate, glabrous	4.5–5 mm long, lanceolate, acuminate, glabrous
Dorsal sepal	3.7 x 1.5 mm, oblong-elliptic, obtuse, 1-veined	3–4.5 x 1.1–2 mm, elliptic-ovate to oblong ovate, subacute, 1-veined	3–3.8 x 1.1–1.5 mm, oblong-elliptic to elliptic-ovate, subobtuse, 3-veined
Lateral sepals	4.2 x 1.3 mm, obliquely elliptic-ovate, subacuminate, subobtuse, 1-veined	3.5–4 x 1.6–2 mm, oblong ovate, subacute, 1-veined	3–4 x 1.3–2 mm, obliquely ovate to elliptic-ovate, slightly concave at the base, subacuminate, subobtuse, 3-veined
Petals	3.2 x 1 mm, obliquely lanceolate-ovate, obtuse, 1-veined, ciliate in the basal 2/3	2.8–4 x 0.4–0.8 mm, narrowly linear to narrowly ligulate, somewhat falcate, subobtuse, 1-veined, margins sparsely pilose	2.8–3.2 x 0.4–0.8 mm, obliquely linear-oblanceolate to linear-ligulate, apex obtuse to truncate, 1-veined, ciliate along margins
Lip shape	2.4 x 2 mm, concave in the lower part, subsessile, suborbicular-obovate, apex apiculate	2.5–3.5 x 2–2.8 mm, gibbose at the base, cochleate above, shortly unguiculate, obovate in outline, rounded at apex	2.5–3.2 x 2–2.6 mm, concave in the lower part, subsessile, suborbicular-obovate, apex rounded
Lip disc	with 3 thickened, dendritic branching veins	with 3 thickened, dendritic branching veins	with 3 thickened, dendritic branching veins

Specimen *Bastian 937* is heterotypic. Two specimens from this collection, which are currently included in the general collection of LPB, are *C.maldonadoana*. Plants deposited in the boxes with the undetermined materials in the same herbarium fit the characteristic of *C.badia* Renz *ex* Kolan. & Szlach.

#### 11. Cranichis mandonii

Taxon classificationPlantaeAsparagalesOrchidaceae

Schltr., Repert. Spec. Nov. Regni Veg. Beih. 10: 38. 1922.

B1724F72-714F-593F-A8BC-1C4BB03DCED4

##### Type.

BOLIVIA. *Mandon 1163* (lectotype, designated by Szlachetko and Kolanowska (2019: 12): AMES!; isolectotypes, BM, G, GH, NY, P, S).

##### Diagnosis.

**Figure 13.**
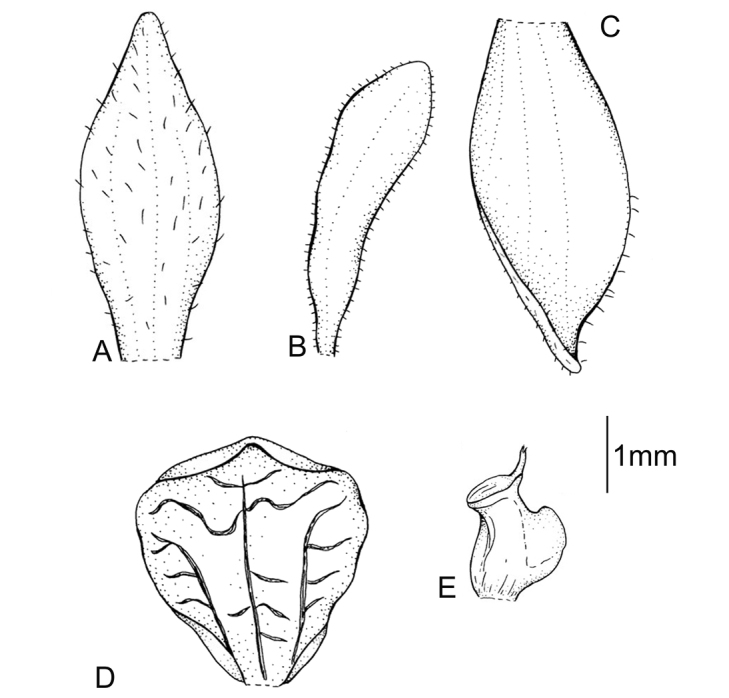
*Cranichismandonii* Schltr. **A** Dorsal sepal. **B** Petal. **C** Lateral sepal. **D** Lip. **E** Gynostemium. Redrawn by A. Król from Garay’s illustration of specimen collected by *Mandon 1163* (AMES).

Plants 40–60 cm tall. Leaves 1–2, basal, petiolate; petiole 13–16 cm long, narrow, canaliculate; blade 6–8.5 cm long, 3.7–4.4 cm wide, oblong to ovate, slightly oblique, base cordate. Scape delicate, terete, enclosed in 5-6 acuminate sheaths, apically glandular-pilose. Inﬂorescence 12 cm long, cylindrical, subdensely many-ﬂowered. Flowers with tepals maroon at base, white at apex. Floral bracts about 5–5.5 mm long, narrowly lanceolate, acuminate. Pedicellate ovary about 5–5.5 mm long, fusiform- cylindrical, sparsely glandular. Sepals sparsely pubescent on the outer surface. Dorsal sepal 4.5 mm long, 1.5 mm wide, lanceolate-ovate to oblong ovate, subacute to subobtuse, 3-veined. Petals 4 mm long, 1 mm wide, oblong ligulate to oblong oblanceolate, obtuse to rounded at apex, 1-veined, margins minutely ciliate-papillate. Lateral sepals 4.5 mm long, 2 mm wide, elliptical to elliptic-ovate, subacute, 3-veined. Lip 3 mm long and wide, basally gibbose, subsessile, suborbicular-obovate to suborbicular, apically rounded; disc 3-veined, veins somewhat thickened, dendritic branching, without any nodules. Gynostemium 2 mm long. Fig. 13.

##### Habitat and ecology.

Terrestrial plants growing in Yungas montane cloud forest at altitudes between 2600-3100 m. Flowers in April and May.

##### Representative specimens.

**BOLIVIA. Larecaja**: Sorata, 2650–3100 m. Apr–May 1860. *G. Mandon 1163* (AMES!, BM, G, GH, NY, P, S). Fig. 2.

##### Notes.

*Cranichismandonii* is often considered as conspecific with *C.ciliata* (e.g. Schweinfurth 1958), however, the two species differ in the ornamentation on the sepals, which in *C.mandonii* is sparsely pubescent and in *C.ciliata* glabrous.

#### 12. Cranichis muscosa

Taxon classificationPlantaeAsparagalesOrchidaceae

Sw., Prodr. 120. 1788.

2CE747C0-0CCA-504C-B832-E695FD953442

Cranichis ovata Wikstr., Kongl. Vetensk. Acad. Handl. 73. 1920.

##### Type.

JAMAICA. *s.n*. (lectotype, designated by Garay (1978: 202): BM!; isolectotypes, LD, S!, W!).

##### Diagnosis.

**Figure 14.**
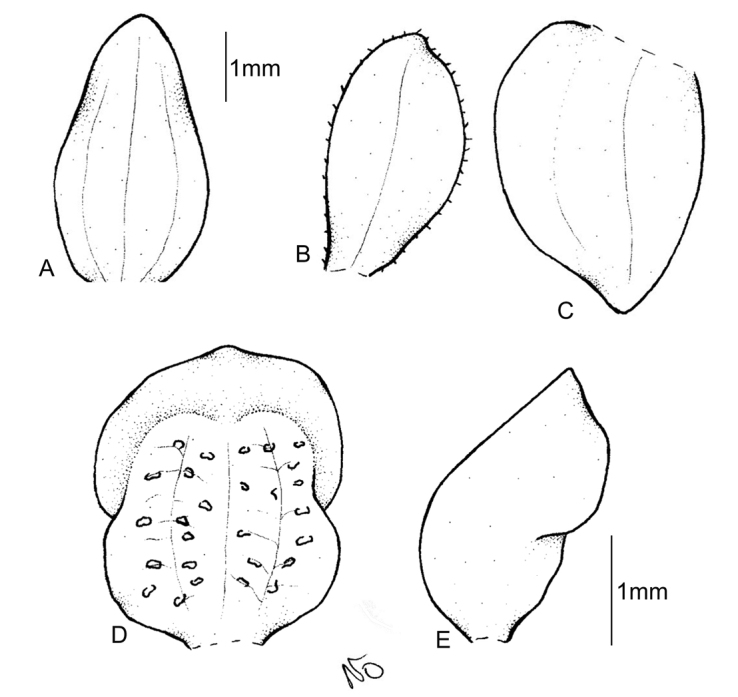
*Cranichismuscosa* Sw. **A** Dorsal sepal. **B** Petal. **C** Lateral sepal. **D** Lip, front view. **E** Lip, side view. Drawn by N. Olędrzyńska from *Garay & Sweet 1057* (AMES).

Plants up to 25 cm tall, erect, strict or ﬂexuose. Leaves 3–5, basal, rosulate, petiolate; petiole 2-3 cm long; blade 2.5–3 cm long, up to two cm wide, ovate, elliptic-ovate to oblong, acute to subobtuse. Scape slender, enclosed in ﬁve sheaths. Inﬂorescence up to 7.5 cm long, cylindrical, subdensely many-ﬂowered. Flowers small, white. Floral bracts four to ﬁve mm long, lanceolate to ovate-lanceolate, acuminate. Pedicellate ovary 5-6 mm long, glabrous. Dorsal sepal 2.2 mm long, one mm wide, oblong-lanceolate to oblong-ovate, acute, 3-veined. Petals 2 mm long, 0.6 mm wide, linear-ligulate to narrowly oblanceolate, obtuse, subfalcate, margins ciliate, 1-veined. Lateral sepals 3 mm long, 1.8 mm wide, obliquely oblong-ovate to elliptic-ovate, acuminate, acute, obscurely 2-veined. Lip 2.2 mm long, 1.87 mm wide, concave, subsessile, ovate to suborbicular-ovate, shortly apiculate to acute; disc with irregular knob-like projections in the centre. Gynostemium 2 mm long. Fig. 14.

##### Habitat and ecology.

According to Vásquez et al. (2014) this species grows as an epiphyte in humid amazon forest at altitudes between 500–1000 m. Flowers in April.

##### Representative specimen.

**BOLIVIA. Santa Cruz**: Velasco. Parque Nacional Noel Kempff Mercado. Campamento Las Gamas. Bosque de colina, 900 m. 4 April 1993. *T. Killeen et al. 5050* (herb. Vásquez, MO, USZ–Vásquez et al. 2014). Fig. 2.

##### Notes.

This is a widely distributed species. Its geographical range extends from USA (Florida) to Brazil and Bolivia. It is recognized by its foliaceous scape, minutely ciliolate petals and lip with membranous margin.

#### 13. Cranichis pulvinifera

Taxon classificationPlantaeAsparagalesOrchidaceae

Garay, Fl. Ecuador 9: 204. 1978.

E52DCC0C-60E8-521E-BE73-9DC65050335F

##### Type.

COLOMBIA. *Bristol 1227* (holotype AMES!).

##### Diagnosis.

**Figure 15.**
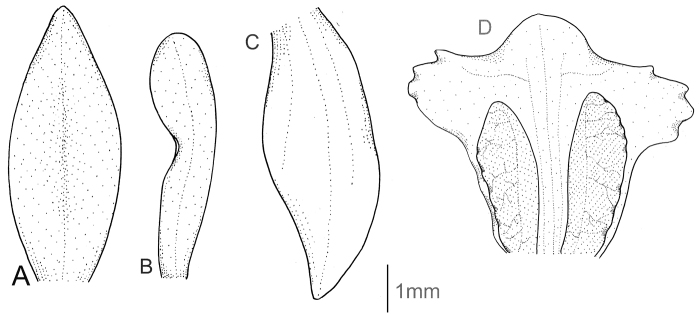
*Cranichispulvinifera* Garay. **A** Dorsal sepal. **B** Petal. **C** Lateral sepal. **D** Lip. Drawn by P. Baranow from *M. López & G. Villegas 74* (LPB).

Plants 42 cm tall. Leaves 5, basal, petiolate; petiole 4–6.5 cm long, narrow; blade 4–6 cm long, 2.2–3 cm wide, ovate to elliptical, acute, with rounded base. Scape erect, slender, ca. 5-sheathed, the lowermost foliaceous. Inﬂorescence 8 cm long, cylindrical, loosely many-ﬂowered. Flowers with beige sepals, salmon petals and brown lip, greenish. Floral bracts up to 8 mm long, lanceolate, acuminate, glabrous. Pedicellate ovary 9–11 mm long, glabrous. Dorsal sepal 5.1 mm long, 2 mm wide, narrowly elliptical, obtuse, l-veined. Petals 4.5 mm long, 1.3 mm wide, linear-ligulate, rounded, l-veined, glabrous. Lateral sepals 5.3 mm long, 2.2 mm wide, obliquely ovate-elliptical, obtuse, obscurely 3-veined. Lip 4.2 mm long, 4.5 mm wide, navicular or subsaccate, sessile, triangular-obovate, truncately 3-lobed in front with triangular, obtuse middle lobe and ovate, erose lateral lobes; disc with a pair of inﬂated cushions which are sparsely covered with large papillae. Gynostemium 2 mm long. Fig. 15.

##### Habitat and ecology.

Terrestrial in humid montane forest. It grows at an altitude of ca. 2400 m. Flowers in April. According to Vásquez et al. (2014) this species grows in the Yungas ecoregion at altitudes between 2000-3000 m.

##### Representative specimen.

**BOLIVIA. La Paz**: Prov. Nor Yungas. Parque Nacional Cotapata. Bajada Hornuni. 16°12’40”S 67°53’26”W, 2420 m. 5 April 2006. *M. López & G. Villegas 74* (LPB!). Fig. 2.

##### Notes.

This species is recorded in Colombia, Ecuador and Bolivia. Surprisingly, so far, it has not been reported from Peru.

#### 14. Cranichis silvicola

Taxon classificationPlantaeAsparagalesOrchidaceae

Renz ex Kolan. & Szlach., Nordic J. Bot. 32(3): 296. 2014.

3AF3F9E1-15FD-542F-B40D-1934B7F73C8E

##### Type.

VENEZUELA. *Renz 6139* (holotype RENZ!).

##### Diagnosis.

**Figure 16.**
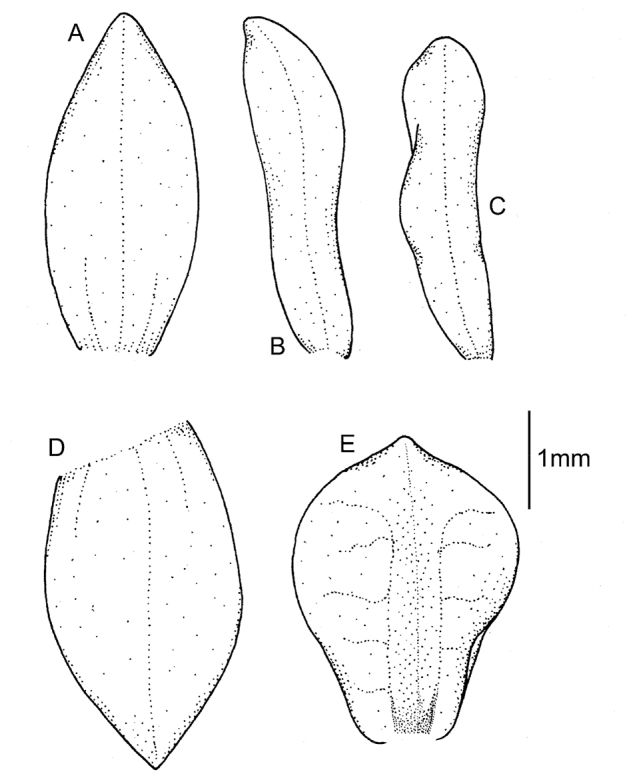
*Cranichissilvicola* Renz *ex* Kolan. & Szlach. **A** Dorsal sepal. B-**C** Petal. **D** Lateral sepal. **E** Lip. Drawn by P. Baranow from *A. Fuentes & A. Macacha 16283* (LPB).

Plants 34–40 cm tall. Leaves 1–2, basal, petiolate; petiole 4–12 cm long, narrow, canaliculate; blade 6–22 long, 2.4–6 cm wide, elliptical, shortly acuminate, cuneate at base. Scape enclosed in 4-5 non-foliaceous sheaths, microscopically ciliate on upper half. Inﬂorescence 5(18) cm long, cylindric-conical, rather laxly many-ﬂowered. Flowers white with green veins and lip, glabrous. Floral bracts 3.4–5 mm long, lanceolate to ovate lanceolate, acute or acuminate, sparsely glandular to almost glabrous. Pedicellate ovary up to 5.5 mm long, glabrous. Dorsal sepal 3.0–3.5 mm long, 1.5–1.7 mm wide, narrowly elliptic-obovate, obtuse, 3-veined. Petals 3.4–3.5 mm long, 0.8–1.0 mm wide, oblong-ligulate to linear-oblanceolate, obtuse, 1-veined, glabrous. Lateral sepals 3.0–3.5 mm long, 1.5–2.0 mm wide, obliquely oblong-ovate to ovate-elliptical, obtuse, 3-veined (sometimes obscurely 3-veined). Lip about 3.0 mm long, 2.1–2.2 mm wide, cochleate, subsessile, from the cuneate base suborbicular to broadly obovate, subacute at apex; disc with 3 veins, lateral veins branching, middle vein sometimes only extends to the middle part of the lip. Gynostemium 1.8–2 mm long. Fig. 16.

##### Habitat and ecology.

Terrestrial plants growing in Yungas montane humid forest with *Weinmannia* L. (Cunoniaceae) and *Miconia* Ruiz & Pav. (Melastomataceae), and in cloud forest at an altitude of ca. 3088 m. Also reported from a lower altitude of 2100 m. Flowers in April and December.

##### Representative specimens.

**BOLIVIA. La Paz**: Prov. B. Saavedra. Area Natural de Manejo Integrado Apolobamba, Paján, sector Cochapata. 15°07’13”S 68°53’45”W, 3088 m. 22 April 2010. *A. Fuentes & A. Machaca 16283* (LPB!). **Tarija**: Prov. Cercado. Cerca Victoria, 2180 m. 24 December 1985. *E. Bastian 265* (LPB!). Fig. 2.

##### Notes.

This species resembles *C.badia*, but differs in having a subsessile, cuneate lip base (unguiculate in *C.badia*) and floral bracts equal in length to pedicellate ovary (vs. much shorter). Unlike in the type material the margins of the lip of Bolivian specimens are not undulate.

#### 15. Cranichis stictophylla

Taxon classificationPlantaeAsparagalesOrchidaceae

Schltr., Repert. Spec. Nov. Regni Veg. Beih. 7: 62. 1920.

DF4509B5-E287-5F22-9091-E82811E88C35

##### Type.

COLOMBIA. *Madero s.n.* (B†).

##### Diagnosis.

**Figure 17.**
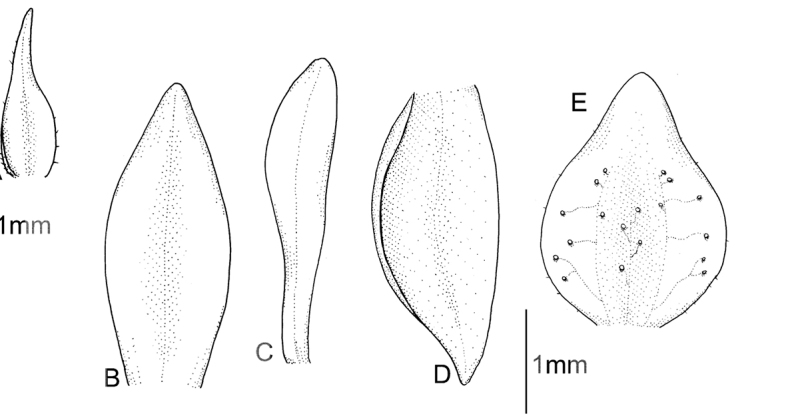
*Cranichisstictophylla* Schltr. **A** Floral bract. **B** Dorsal sepal. **C** Petal. **D** Lateral sepal. **E** Lip. Drawn by P. Baranow from *M. Nee 40653* (LPB).

Plants 33–36 cm tall. Leaf 1, basal, petiolate; petiole 3–4 cm long; blade 4.5–6.5 cm long, 2–3.5 cm wide, ovate, acuminate, base subrounded-cuneate, white spotted. Scape glandulous-pilose toward the apex, enclosed in 5–6 sheaths. Inﬂorescence 3–6 cm long, cylindrical, sublaxly to subdensely 15-ﬂowered. Flowers greenish-white, glabrous. Floral bracts 2.1–4 mm long, ovate-lanceolate, acuminate, sparsely glandular. Pedicellate ovary 7–7.5 mm long, densely glandular-ciliate. Dorsal sepal 2.9–3 mm long, 0.8–1.2 mm wide, oblong elliptical, obtuse, 1-veined. Petals 2.9–3 mm long, 0.7–0.8 mm wide, obliquely linear-oblanceolate, obtuse, glabrous, 1-veined. Lateral sepals 3 mm long, 1.2–1.5 mm wide, obliquely elliptic-ovate, slightly concave at base, subacuminate, obtuse, obscurely 2-veined. Lip 2.5 mm long, 2.0–1.9 mm wide, slightly concave, sessile, ovate, rounded or obtuse; disc with 3 or 5 dendritic thick branching veins, with prominent nodules. Gynostemium 1–1.3 mm long. Fig. 17.

##### Habitat and ecology.

Terrestrial in Tucumano-Boliviano cloud forest and disturbed forest with *Prumnopitys* Phil. (Podocarpaceae), Myrtaceae, *Dicksonia* L’Hér. (Dicksoniaceae, and *Cyathea* Kaulf. (Cyatheaceae) at altitudes between 2100-2200 m. Flowers in June.

##### Representative specimens.

**BOLIVIA. Santa Cruz**: Prov. Florida, 7 km NE of Mairana. Southern limit of expanded Parque Nacional Amboró, entering from Mairana, 2200 m. 2 June 1991. *M. Nee 40653* (LPB!), Prov. Vallegrande, 5 km de Loma Larga a Vallegrande. 7 June 1996. *M. Kessler et al. 6352* (LPB!). Fig. 2.

##### Notes.

This species is often considered as conspecific with *C.diphylla* (e.g. Garay 1978) from which it differs, e.g., in having a 1-veined dorsal sepal.

### Excluded species

#### Cranichis multiflora

Taxon classificationPlantaeAsparagalesOrchidaceae

(Poepp. & Endl.) Cogn., Fl. Bras. 3(4): 248. 1895.

A4B3ABBA-23FA-59A2-ABEB-06EED53BC101

##### Type.

PERU. *Poeppig 1724* (?). *Ponthievamultiflora* Poepp. & Endl., Nov. Gen. Sp. Pl. 2: 16, t. 123. 1838.

##### Notes.

This species was included by Vásquez et al. (2014) in their list of Bolivian orchids as a species of *Cranichis*, however, the original illustration of *Ponthievamultiflora* shows that the petal, gynostemium and lip are basally fused, a character not recorded for *Cranichis*, but is present in *Ponthieva.* Unfortunately, we were not able to find the specimen *Vásquez C. et al. 670* to confirm its generic placement in *Ponthieva*.

#### Cranichis polyantha

Taxon classificationPlantaeAsparagalesOrchidaceae

Schltr., Repert. Spec. Nov. Regni Veg. Beih. 7: 61. 1920.

959D8438-6680-5077-AD2C-D776636C8E5C

##### Type.

COLOMBIA. *Madero 22* (B†, lectotype, designated by (Garay 1978: 203): AMES!–drawing).

##### Notes.

**Figure 18.**
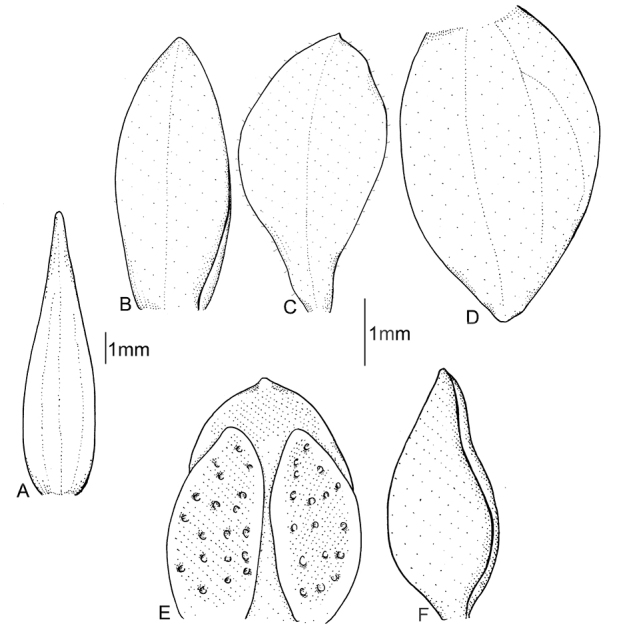
*Cranichis* sp. **A** Floral bract. **B** Dorsal sepal. **C** Petal. **D** Lateral sepal. **E** Lip, front view. **F** Lip, side view. Drawn by P. Baranow from *I. Jimenez 5547 et al.* (LPB).

We examined *Jimenez 5547 et al.* (LPB) cited as reference material for this species in Bolivia by Jiménez-Pérez (2011) and Vásquez et al. (2014) and in our opinion it is not *C.polyantha* (Fig. 18).

The only information about the original collection of this species is the incomplete illustration deposited in AMES. While Schlechter (1920) did not describe any ornamentation on the petals or lip of *C.polyantha*, both Garay (1978) and Bennett and Christenson (1995) identified this species based on its ciliate petals and lip covered with numerous nodules or papillose-verrucose.

### Incertae sedis

*I. Jimenez 5547 et al.* (LPB!): this specimen was cited by Jiménez-Pérez (2011) as *C.polyantha*, however, several characters of this specimen do not fit the morphological characteristic of this species (Fig. 18). In *Jimenez 5547 et al.* the lip is covered with small nodules and the margins of the petals are glandular-ciliate. Currently, with only a single specimen in LPB and discrepancies between the original description of *C.polyantha* and the treatment of this taxon by Garay (1978) and Bennett and Christenson (1995), we prefer not to describe a new species based on this specimen.

## Discussion

As summarized by Mace (2004) and Dubois (2003) effective conservation of species requires a good taxonomic basis. Unfortunately, the number of taxonomic studies that estimate the actual (alpha) diversity of ecosystems is declining (Wägele et al. 2011). Vásquez et al. (2003) note that there is a need for further research on orchids in Bolivia as the actual number of Orchidaceae occurring in this country is most probably much higher than currently recorded. In this paper we provide the most complete data on Bolivian *Cranichis* published so far. The species characteristics and dichotomous key presented will simplify the process of identification of *Cranichis* by local botanists and improve the quality of regional checklists.

Based on this study, details of perianth segments are the most useful diagnostic characters in *Cranichis*, however, the proper identification of some taxa is difficult without information about leaf petiole size (*C.longipetiolata, C.polyantha*) or inflorescence shape (*C.lehmannii, C.cylindrostachys*). Only two Bolivian *Cranichis* (*C.garayana, C.pulvinifera*) are characterized by a distinctly 3-lobed lip with irregularly erose to erose-lancinate margins. The lip form and ornamentation, together with shape, size and ornamentation on the margins of the tepals, can be used to identify most species in this genus in Bolivia. Petals of Bolivian *Cranichis* are 1-veined, whereas sepals can be 1-, 2-, 3- or 5-veined. Tepals can be glabrous or variously ciliate, papillate or pilose. While extensive studies on *Cranichis* (e.g. Kolanowska and Szlachetko 2014; Szlachetko and Kolanowska 2019) indicate that the number of veins is constant within a species we noted a variation in venation in Bolivian populations, which correspond to *C.badia.* There is a possibility that they represent an undescribed species of *Cranichis*, but we prefer not to delimit any new species based solely on the number of veins.

Similar to many other new species of orchids (e.g. Averyanov et al. 2015; Baquero et al. 2018; Lin et al. 2020) the new taxa described here are based on single specimens. This is not surprising as numerous tropical orchids are local endemics and their geographical ranges are often limited to small patches of forests or single valleys (Koopowitz 1992; Vermeulen and Lamb 2011). Among the numerous factors that affect the geographical distribution and diversity of orchids (Dodson 2003), local radiations resulting in the evolution of numerous species of orchids in relatively small areas seems to be the most important (e.g. Jost 2004). Describing new species based on limited data is obviously risky as there is no information on the intraspecific variation in such taxa. However, considering the ongoing loss of habitats for orchids (Wraith et al. 2020) it is important to identify novel orchids before they go extinct (Swarts and Dixon 2009; Vermeulen et al. 2014), even though based on incomplete data on their morphological variation.

## Conclusions

Here we present synopsis of Bolivian species of *Cranichis.* Morphological characteristics of all the species together with the illustrations of the perianth segments and the identification key will be useful for local botanists collecting orchids. The proper documentation of the distribution of rare and endangered orchids in Bolivia will help in the establishment of more advanced nature management programs.

The occurrence of 12 species of *Cranichis*, including two new species and two new records, in Bolivia was confirmed. We did not validate the presence of *C.diphylla, C.lehmannii* and *C.muscosa* in this country. Moreover, in our opinion, the previously published Bolivian record for *C.polyantha* is doubtful and the specimen collected is an undescribed species. However, due to incongruities between the original description of *C.polyantha* and concept of this orchid presented by Garay (1978) and Bennett and Christenson (1995), we prefer not to delineate a new taxon.

## Supplementary Material

XML Treatment for Cranichis atrata

XML Treatment for Cranichis badia

XML Treatment for Cranichis beckii

XML Treatment for Cranichis ciliata

XML Treatment for Cranichis cylindrostachys

XML Treatment for Cranichis diphylla

XML Treatment for Cranichis garayana

XML Treatment for Cranichis lehmannii

XML Treatment for Cranichis longipetiolata

XML Treatment for Cranichis maldonadoana

XML Treatment for Cranichis mandonii

XML Treatment for Cranichis muscosa

XML Treatment for Cranichis pulvinifera

XML Treatment for Cranichis silvicola

XML Treatment for Cranichis stictophylla

XML Treatment for Cranichis multiflora

XML Treatment for Cranichis polyantha

## Supplementary materials

Supplementary material 1 Annex1 Data type Collections list Explanation note Herbarium collection list https://binary.pensoft.net/file/616521This dataset is made available under the Open Database License (http://opendatacommons.org/licenses/odbl/1.0/). The Open Database License (ODbL) is a license agreement intended to allow users to freely share, modify, and use this Dataset while maintaining this same freedom for others, provided that the original source and author(s) are credited.Kolanowska et al.
