# ﻿Corrigendum: Kolanowska M, Baranow P, Nowak S, Fuentes A (2021) Materials to the revision of the genus *Cranichis* (Orchidaceae) in Bolivia. PhytoKeys 186: 11–41. https://doi.org/10.3897/phytokeys.186.71499

**DOI:** 10.3897/phytokeys.192.81571

**Published:** 2022-03-14

**Authors:** Marta Kolanowska, Przemysław Baranow, Sławomir Nowak, Alfredo Fuentes

**Affiliations:** 1 University of Lodz, Faculty of Biology and Environmental Protection, Department of Geobotany and Plant Ecology, Lodz, Poland University of Lodz Lodz Poland; 2 Department of Biodiversity Research, Global Change Research Institute AS CR, Brno, Czech Republic Department of Biodiversity Research, Global Change Research Institute AS CR Brno Czech Republic; 3 Department of Plant Taxonomy and Nature Conservation, University of Gdańsk, Gdańsk, Poland University of Gdańsk Gdańsk Poland; 4 Herbario Nacional de Bolivia, Instituto de Ecología, Universidad Mayor de San Andrés, La Paz, Bolivia Universidad Mayor de San Andrés La Paz Bolivia

In our recent work (Kolanowska et al. 2021), a wrong figure was published as *Cranichisbeckii* Kolan., Baranow, S. Nowak & A. Fuentes, sp. nov. Instead of the newly described species, the figure of *Cranichisbadia* was presented twice. Here, we present the correct illustration of *C.beckii*.

**Figure 1.**
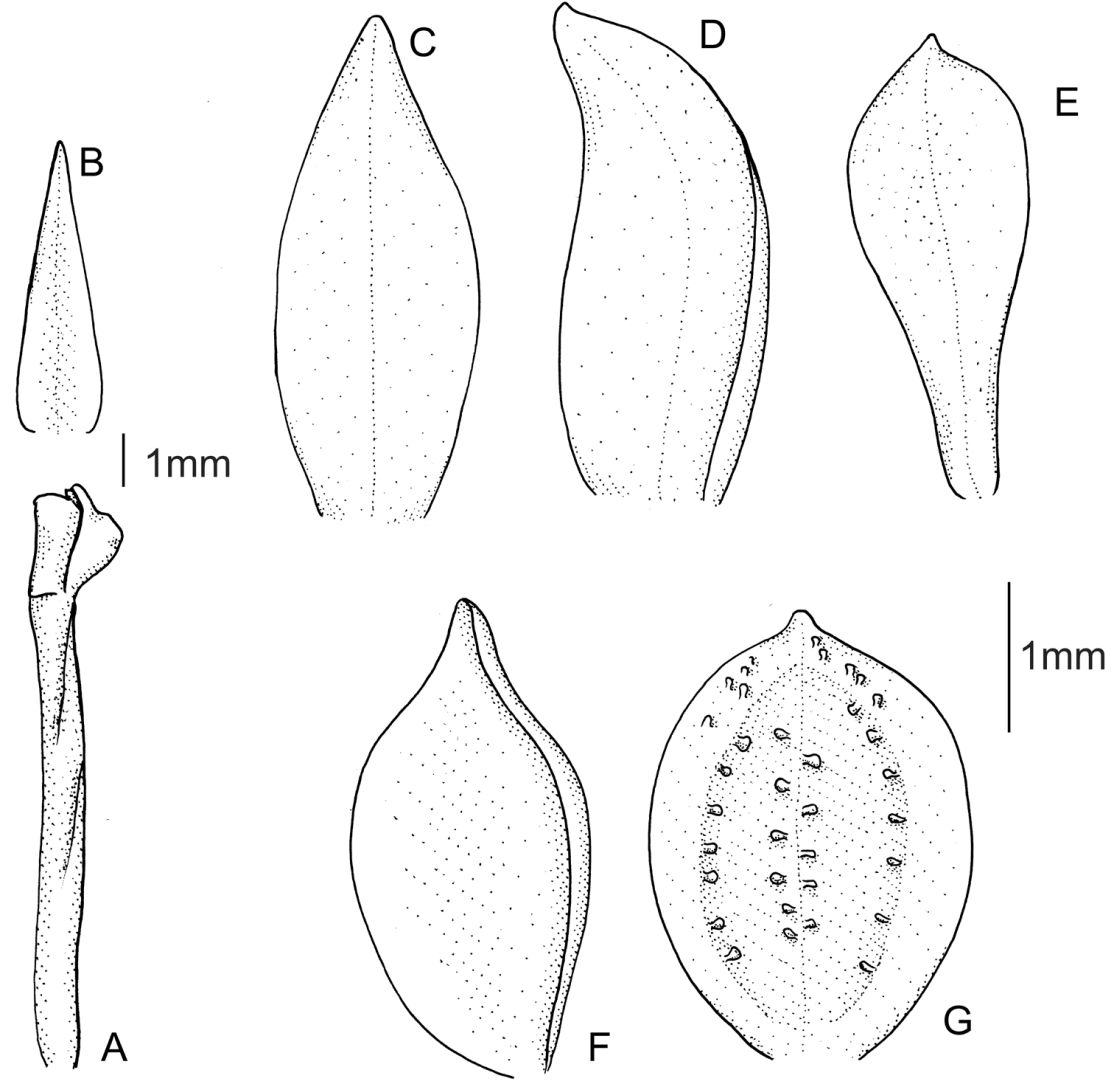
*Cranichisbeckii* sp. nov. **A** ovary and gynostemium **B** floral bract **C** dorsal sepal **D** lateral sepal **E** petal **F** lip, side view **G** lip, front view. Drawn by P. Baranow from S. G. Beck 313 et al. (LPB).
