# Supplementary material for: Materials to the revision of the genus Cranichis (Orchidaceae) in Bolivia
Source: PhytoKeys. 2021 Nov 29;186:11–41. doi: 10.3897/phytokeys.186.71499 (PMC8648718; doi:10.3897/phytokeys.186.71499)
Supplement: Supplementary material 1 — Annex1 [file phytokeys-186-011-s001.pdf]

Representatives of Bolivian *Cranichis* species examined in this study.

Species	Collector & collection nr	Herbarium acronym
Cranichis atrata	R. Vasquez et al. 1429	LPB
Cranichis atrata	E.P. Killip et al. 39868	US
Cranichis atrata	H. Rimann s.n.	W
Cranichis atrata	E. Killip & A.C. Smith 20643	AMES
Cranichis atrata	M. Ospina H. & J. Idrobo 146	AMES
Cranichis atrata	C. H. Dodson et al. 17035	RPSC
Cranichis atrata	N. Fassett 25804	AMES
Cranichis atrata	E.P. Killip & A.C. Smith 16730	AMES, US
Cranichis badia	L. Cayola et al. 3657	LPB
Cranichis badia	E. Bastian 937	LPB
Cranichis badia	M. Schneider 172/5	RENZ
Cranichis badia	Y. Figueroa-C., J. Mora & T. Vivas 867	COL
Cranichis badia	M. Schneider 11/1	COL
Cranichis badia	M. Schneider 253	COL
Cranichis badia	J. Ordonez et al. 1566	COL
Cranichis badia	F. C. Lehmann 8173	K
Cranichis badia	F. C. Lehmann 1 or s.n.	W
Cranichis badia	C. Dodson 98	RPSC
Cranichis badia	G. Harling et al. 8831	AMES
Cranichis badia	C. Dodosn & A. Hirtz 15816	RPSC
Cranichis badia	A. Hirtz et al. 2490	RPSC
Cranichis badia	D. Dallesandro 0390	RPSC
Cranichis badia	M. E. André 2222	K
Cranichis badia	H. G. Barclay & P. Juajibioy 9933	COL
Cranichis badia	O. Renz 6174	RENZ
Cranichis badia	O. Renz 6175	RENZ
Cranichis beckii	S. G. Beck 313 et al.	LPB
Cranichis ciliata	B. Ollgaard et al.. 74391	QCNE
Cranichis ciliata	G. P. Lewis 3263	QCNE
Cranichis ciliata	C. H. Dodson & A. Hirtz 15815	QCNE
Cranichis ciliata	C. H. & T. A. Dodson 16414	QCNE
Cranichis ciliata	P. Ibish & C. Ibish 96.0020	LPB
Cranichis ciliata	M. Kessler et al. 9412	LPB
Cranichis ciliata	J. C. Solomon 9678	LPB
Cranichis ciliata	A. Fuentes & R. Rodas 16115	LPB
Cranichis ciliata	I. Loza et al. 1621A	LPB
Cranichis ciliata	I. Loza et al. 1698	LPB
Cranichis ciliata	A. Fuentes & D. Alanes 15018	LPB
Cranichis ciliata	A. Fuentes 2903	LPB
Cranichis ciliata	F. C. Lehmann 7258	AMES, NY
Cranichis ciliata	F. C. Lehmann 8155	AMES, NY
Cranichis ciliata	F. C. Lehmann 1	W
Cranichis ciliata	E.L. Core 1020	US
Cranichis ciliata	J.J. Triana 621	P, US
Cranichis ciliata	F. Silverstone-Sopkin et al. 3817	CUVC
Cranichis ciliata	G. Mandon 1163	W
Cranichis ciliata	J. Macbride 4045	W
Cranichis ciliata	A. Hirtz s.n.	RPSC

Cranichis ciliata	A. Hirtz 1335	RPSC
Cranichis ciliata	A. Hirtz 1593	RPSC
Cranichis ciliata	A. Hirtz 2583	RPSC
Cranichis ciliata	R. Spruce 5216	W
Cranichis ciliata	W. Jon s.n.	W
Cranichis ciliata	F. C. Lehmann 470	W
Cranichis ciliata	H. Karsten s.n.	W
Cranichis ciliata	A. J. Perez et al. 8907	QCA
Cranichis ciliata	C. Dodson et al. 10726	QCA
Cranichis ciliata	A. Humboldt s.n.	W
Cranichis cylindrostachys	F. Miranda et al. 1236	LPB
Cranichis cylindrostachys	B. MacBryde 579	AMES
Cranichis diphylla	M. Mites et al. 431	QCNE
Cranichis diphylla	M. Mites et al. 457	QCNE
Cranichis diphylla	O. Swartz s.n.	AMES, BM, W
Cranichis diphylla	R. Fonnegra et al. 5266	MO
Cranichis diphylla	H. Rby & F. Pennell 944	AMES
Cranichis diphylla	E. Killip & A. Smith 15946	AMES
Cranichis diphylla	E. Killip & A. Smith 16060	AMES
Cranichis diphylla	K. Cremers 7408	CAY, P
Cranichis diphylla	J. Hawkins 1812	MO
Cranichis diphylla	L. Williams & A. Alston 268	BM
Cranichis diphylla	F. C. Lehmann 1772 BM	BM
Cranichis diphylla	H. Harris 7555 BM	BM
Cranichis garayana	I. Jimenez & F. Miranda 3854	LPB
Cranichis lehmannii	R. Callejas et al. 9899	NY
Cranichis lehmannii	R. Callejas et al. 8674	NY
Cranichis lehmannii	R. Callejas et al. 9541	NY
Cranichis lehmannii	J. L. Zarucchi & F. Roldan 7262	MO
Cranichis lehmannii	P. M. Jorgensen et al. 61322	QCNE
Cranichis lehmannii	F. C. Lehmann 77	W
Cranichis longipetiolata	A. Sagdstegui et al. 15251	QCNE
Cranichis longipetiolata	M. Mendoza & S. Acebo 912	LPB
Cranichis longipetiolata	Parada et al. 4208	LPB
Cranichis longipetiolata	R. Arevalo et al. 787	COL
Cranichis longipetiolata	R. Ferreyra 3120	AMES
Cranichis longipetiolata	A. Hirtz 2651	RPSC
Cranichis maldonadoana	E. Bastian 937	LPB
Cranichis mandonii	G. Mandon 1163	AMES
Cranichis mandonii	Y. Figueroa et al. 880	COL
Cranichis mandonii	G. Mandon 1163	AMES
Cranichis muscosa	L. Garay & H. Sweet 1057	AMES
Cranichis muscosa	J. Betancur 8748	COL
Cranichis muscosa	C. Wright 620 & s.n.	W
Cranichis muscosa	E. Poeppig s.n.	W
Cranichis muscosa	L. Hahn 90	W
Cranichis muscosa	W. Morris s.n.	W
Cranichis muscosa	G. P. Lewis 3098	QCNE
Cranichis muscosa	O. Swartz s.n.	BM, W
Cranichis polyantha	O. Renz 4126	AMES

Cranichis polyantha	M. Madero 22	AMES
Cranichis polyantha	Fernandez P. 5882	COL
Cranichis polyantha	O. de Benavides 9814	COL
Cranichis polyantha	M. Schneider 615	COL
Cranichis polyantha	W. Hodge 6137	AMES
Cranichis polyantha	A. Hirtz 2156	RPSC
Cranichis polyantha	C. Dodson 221	RPSC
Cranichis polyantha	A. Hirtz et al. 2343	RPSC
Cranichis polyantha	C. Dodson et al. 10626	QCA
Cranichis polyantha	A. Hirtz 2421	RPSC
Cranichis polyantha	H. van der Werff & W. Palacios 10467	QCNE
Cranichis pulvinifera	M. López & G. Villegas 74	LPB
Cranichis pulvinifera	M. Bristol 1227	AMES
Cranichis pulvinifera	D. Dalessandro 623	RPSC
Cranichis pulvinifera	C. Luer et al. 498	RPSC
Cranichis silvicola	A. Fuentes & A. Machaca 16283	LPB
Cranichis silvicola	E. Bastian 265	LPB
Cranichis silvicola	M. Cordoba et al. 3143	COL
Cranichis silvicola	F. C. Lehmann 8505	K, RENZ
Cranichis silvicola	C. Sheviak et al. CS825	AMES
Cranichis silvicola	O. Renz 6141	RENZ
Cranichis sp.	I. Jimenez 5547 et al.	LPB
Cranichis stictophylla	M. Nee 40653	LPB
Cranichis stictophylla	M. Kessler et al. 6352	LPB
Cranichis stictophylla	F.W. Pennell 7597	AMES, NY
Cranichis stictophylla	P. Hutchison & J. Wright 5792	AMES
